# Family‐Centred Breastfeeding Interventions to Improve Exclusive Breastfeeding: A Systematic Review and Meta‐Analysis

**DOI:** 10.1002/nop2.70669

**Published:** 2026-07-03

**Authors:** Ziyang Xie, Xuanli Zhang, Yunyi Zhang, Xiao Xiao

**Affiliations:** ^1^ School of Nursing Southern Medical University Guangzhou China; ^2^ Outpatient and Emergency Department, Shenzhen Maternity and Child Healthcare Hospital Southern Medical University Shenzhen China

**Keywords:** exclusive breastfeeding, family interventions, intervention strategies, meta‐analysis, systematic review

## Abstract

**Aims:**

To explore the effects of different family breastfeeding interventions on improving exclusive breastfeeding rates from 0 to 6 months postpartum and to identify the key characteristics of effective interventions.

**Methods:**

This systematic review and meta‐analysis follows the Cochrane Handbook for Systematic Reviews of Interventions. The risk of bias in the included studies was assessed using the Cochrane Risk of Bias 2 (RoB 2) tool.

**Results:**

Nineteen studies were included in this systematic review. Family‐based interventions significantly improved exclusive breastfeeding rates at 6 months postpartum compared with routine care (OR = 2.93, 95% CI [1.90, 4.50], *p* < 0.001). Interventions underpinned by theoretical frameworks enhanced exclusive breastfeeding within 2 months postpartum compared with non‐theoretical ones (OR = 5.50, 95% CI [2.30, 13.19], *p* < 0.001). Multi‐component interventions were more effective than single‐method approaches (OR = 2.70, 95% CI [2.04, 3.57], *p* < 0.001). Programmes spanning both prenatal and postnatal periods achieved higher exclusive breastfeeding rates at 4–6 months than those implemented in a single phase (OR = 4.84, 95% CI [2.50, 9.40], *p* < 0.001).

**Conclusions:**

Family‐based breastfeeding interventions could improve exclusive breastfeeding rates within the first 6 months postpartum. Future breastfeeding interventions should include key family members, consider the guidance of theoretical frameworks on the intervention design, cover both the prenatal and postnatal periods, and use multiple methods to meet the needs of breastfeeding families.

**Implications for Nursing Practice:**

Nurses, midwives, lactation nurses, and public health nurses should actively involve fathers, grandparents, and other key family members in breastfeeding education and support. Family‐centred breastfeeding interventions may help improve exclusive breastfeeding outcomes and strengthen family support for breastfeeding.

**Registration:**

Not registered.

**Abbreviations**
AAFP: American Academy of Family Physicians
AAP: American Academy of Pediatrics
ACOG: American College of Obstetricians and Gynecologists
BFHI: Baby‐Friendly Hospital Initiative
CDRF: China Development Research Foundation
RoB 2: The Risk of Bias 2
UNICEF: United Nations Children's Fund
WHO: World Health Organization

## 1 Introduction

Breastfeeding is widely recognised as the optimal source of nutrition for infants by global health organisations such as the World Health Organization, the United Nations Children's Fund, and the American Academy of Pediatrics (AAP) (UNICEF 2011; World Health Organization 2015). The AAP recommends exclusive breastfeeding for the first 6 months, with continuation up to 2 years or beyond (American Academy of Pediatrics 2022). Exclusive breastfeeding refers to feeding only breast milk, including expressed milk and essential supplements such as vitamins or medicines (World Health Organization 2018).

Breast milk provides ideal nutrition, enhances immune protection, and reduces the risk of childhood obesity and type 2 diabetes (Aubel et al. 2021; Birukov et al. 2024; Critch et al. 2014; Ho et al. 2018; Horta et al. 2015; Tschiderer et al. 2022). It also strengthens mother–infant bonding and promotes maternal health by reducing postpartum complications and the risk of breast and ovarian cancers, while improving maternal well‐being and reducing postpartum depression (American College of Obstetricians and Gynecologists 2021; Kinsey et al. 2014; Gaitskell et al. 2018; Henshaw 2023; Su et al. 2013; Unar‐Munguía et al. 2017; UNICEF 2018). At the societal level, breastfeeding reduces healthcare costs and environmental burden. A report in The Lancet estimated that optimal breastfeeding could prevent economic losses of up to $302 billion annually (American College of Obstetricians and Gynecologists 2018; Rollins et al. 2016).

Despite these benefits, breastfeeding rates remain suboptimal worldwide. The Global Breastfeeding Scorecard reported that only 40% of infants under 6 months are exclusively breastfed, and in China, the rate was only 29.2%, far below WHO targets (China Development Research Foundation 2019; UNICEF 2017; World Health Organization 2014).

Breastfeeding is influenced by sociocultural, occupational and health‐related factors, as well as the availability of support (Kronborg and Foverskov 2020). Previous breastfeeding promotion strategies have primarily focused on mothers through education, counselling and professional support programmes, with varying effectiveness in improving exclusive breastfeeding outcomes (Galipeau et al. 2018; Kim et al. 2018; Rollins et al. 2016; Wong et al. 2021). In addition, the Baby‐Friendly Hospital Initiative (BFHI), launched by WHO and UNICEF, has been widely implemented and has demonstrated positive effects on breastfeeding initiation and duration (Habte et al. 2025; Pérez‐Escamilla et al. 2016). However, growing evidence suggests that breastfeeding outcomes are also shaped by the broader family environment and the support available to mothers (Bengough et al. 2022; Jusrawati and Suryaningsih 2023; Nie et al. 2023). Increasing evidence highlights the critical role of family members' knowledge and support in breastfeeding initiation and continuation (Nie et al. 2023). Mothers often rely on family members for support, and with increasing multigenerational co‐residence, grandparents—particularly grandmothers—play an important role in breastfeeding outcomes (Chang et al. 2021; Chen et al. 2011; MacDonald et al. 2020; Schrijner and Smits 2018; Wu et al. 2021). Accordingly, WHO and UNICEF have emphasised strengthening social support to improve breastfeeding rates (Schnefke et al. 2023).

Fathers' involvement is also essential for breastfeeding success and child development (American Academy of Family Physicians 2022; de Montigny et al. 2018; Lok et al. 2017; Sherriff et al. 2009). Support from fathers can improve breastfeeding continuation and maternal well‐being (Mithani et al. 2015; Su and Ouyang 2016). However, inappropriate support may undermine maternal autonomy and self‐efficacy (Rempel et al. 2017). The concept of co‐parenting corresponding behaviours emphasises providing support in ways that align with maternal needs and involve shared decision‐making, thereby enhancing breastfeeding outcomes (Davidson and Ollerton 2020).

Co‐parenting refers to parents working together to achieve shared childrearing goals. Feinberg's ecological model describes co‐parenting as involving shared responsibilities, mutual support and coordinated family management (Feinberg 2003). Greater paternal involvement has been associated with longer breastfeeding duration, improved maternal commitment, and reduced parenting stress (Feinberg et al. 2009; Rempel et al. 2020).

Previous systematic reviews have shown that breastfeeding support interventions can improve breastfeeding outcomes and identified several effective intervention components, such as professional support, peer support and multi‐component interventions (Galipeau et al. 2018; Kim et al. 2018; Rollins et al. 2016; Wong et al. 2021). However, less attention has been paid to the role of family members in breastfeeding support. Although fathers, grandparents, and other family members are increasingly recognised as important influences on breastfeeding behaviours, the effectiveness of interventions targeting different family members and the key characteristics of family‐based breastfeeding interventions remain unclear (Aubel 2021; Negin et al., 2016; Sherriff et al., 2014).

Existing family‐based breastfeeding interventions include health education, counselling, family support programmes and breastfeeding discussion groups (Abbass‐Dick et al. 2015; Abbass‐Dick and Dennis 2018; Koh et al. 2021; Ozlüses and Celebioglu 2014). Although these interventions have generally reported positive effects, substantial variation exists in intervention design, delivery methods, timing and targeted family members, making it difficult to determine which intervention approaches and family participants are most effective. Therefore, this systematic review and meta‐analysis aimed to evaluate the effectiveness of family‐based breastfeeding interventions in improving exclusive breastfeeding during the first 6 months postpartum and to identify key intervention characteristics.

## 2 Methods

### 2.1 Design

This systematic review and meta‐analysis followed the approach put forward in the Cochrane Handbook for Systematic Reviews of Interventions (Higgins et al. 2022). The process and outcomes were documented following the guidelines of the Preferred Reporting Items for Systematic Reviews and Meta‐Analyses (PRISMA) (Page et al. 2021). The PRISMA 2020 checklist is provided as Supporting Information.

### 2.2 Search Methods

Studies were selected based on the ‘PICOS’ (Population, Intervention, Comparison, Outcome, Study Design) criteria. Key terms searched were (‘Breast Feeding’ OR ‘Breastfed’ OR ‘Breastfeeding’ OR ‘Breast Fed’ OR ‘Milk Sharing’ OR ‘Exclusive Breast Feeding’ OR ‘Exclusive Breastfeeding’ OR ‘Wet Nursing’ OR ‘Breast Milk’) AND (‘Co‐parenting’ OR ‘Intergeneration Co‐parenting’ OR ‘Parent‐grandparent Co‐parenting’ OR ‘Parenting’ OR ‘Family’ OR ‘Family Support’ OR ‘Multigeneration’ OR ‘Three‐generation’) AND (‘intervention’ OR ‘program’ OR ‘randomized controlled trial’ OR ‘RCT’). The following electronic databases were searched: Cochrane, PubMed, Embase, PsycINFO, Medline, CINAHL, Web of Science and Scopus. The search period was from database inception to November 2025. References from pertinent papers and citations from related meta‐analyses and review articles were also searched.

### 2.3 Inclusion and Exclusion Criteria

#### 2.3.1 Inclusion Criteria

The criteria for the inclusion of literature for this review were: (1) randomised controlled trials or quasi‐experimental studies aimed at improving exclusive breastfeeding rates; (2) breastfeeding interventions implemented within intact family, involving at least one significant family member aged over 18 years with or without the mother (including partners, grandparents, or other cohabiting close relatives); (3) full‐term born healthy infants and singletons; (4) studies that used the exclusive breastfeeding rate as a primary or secondary outcome.

#### 2.3.2 Exclusion Criteria

The following studies were excluded: (1) studies involving participants with substance or drug abuse issues, or who had been incarcerated, separated, or divorced; (2) duplicate publications or studies with incomplete data on exclusive breastfeeding measurements; (3) studies available only as abstracts without full texts; (4) non‐English publications.

### 2.4 Search Outcome

Two researchers independently screened studies according to the PICOS (Population, Intervention, Comparison, Outcome, Study Design) criteria. All retrieved records were imported into EndNote X9 for duplicate removal. Titles and abstracts were screened for eligibility, followed by full‐text review to confirm inclusion.

### 2.5 Quality Appraisal

Risk of bias was independently assessed by two researchers using the Cochrane Risk of Bias 2 (RoB 2) tool. Discrepancies were resolved through discussion and consensus, supported by consultation of the relevant literature where necessary.

### 2.6 Data Abstraction

Data were extracted using structured forms by two independent researchers. Extracted information included country, study objectives, theoretical framework, intervention format, study design, eligibility criteria, sample size, intervention characteristics (content, frequency, duration and timing), data collection points, and outcome measures. Exclusive breastfeeding rates and secondary outcomes were extracted for meta‐analysis, and summary measures, including odds ratios and confidence intervals, were calculated. No data conversion was required. Missing data were narratively reported where applicable.

### 2.7 Synthesis

Data were synthesised quantitatively through meta‐analysis and qualitatively where appropriate. Meta‐analysis was conducted using Stata 13.1 and Review Manager 5.4. Exclusive breastfeeding rate was the primary outcome, and meta‐analysis was performed when at least three studies reported comparable data. Effect sizes were expressed as odds ratios (OR) with 95% confidence intervals (CI). Statistical significance was set at *p* < 0.05, and heterogeneity was assessed using *I* ^2^, with values of < 25%, 25%–49%, and ≥ 50% representing low, moderate and high heterogeneity, respectively (Higgins et al. 2003). A fixed‐effects model was applied when heterogeneity was low; otherwise, a random‐effects model was used.

Subgroup analyses were conducted based on intervention characteristics, including participant type, delivery format, timing, and theoretical framework. Where meta‐analysis was not feasible, findings were summarised descriptively. Sensitivity analyses assessed result robustness, and publication bias was evaluated using Begg and Mazumdar's test and Duval and Tweedie's trim‐and‐fill method (Begg and Mazumdar 1994; Duval and Tweedie 2000).

## 3 Results

A total of 6290 articles were retrieved. Three additional relevant records were identified from a manual search of the reference lists of the included studies, resulting in 6293 records. After removing duplicates, 3717 articles remained.

**FIGURE 1:**
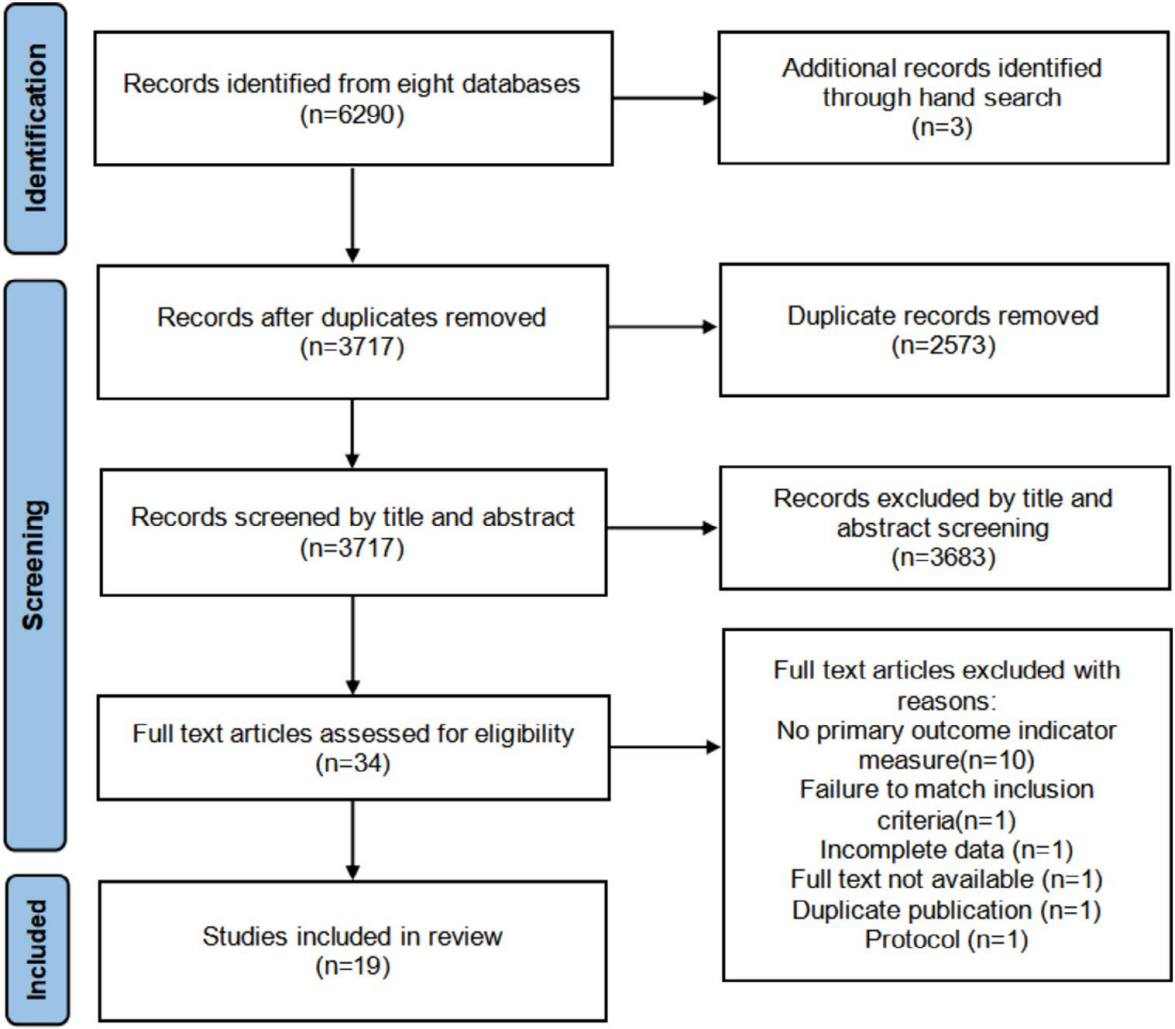
PRISMA flowchart for literature search and selection.

After screening the titles and abstracts of the 3717 articles, 3683 publications were excluded for not meeting the inclusion criteria. The full texts of the remaining 34 articles were checked for eligibility. In the end, there were 10 articles without primary outcome measurements, and 1 each that did not meet the inclusion criteria, had incomplete data, gave no access to the full text, was a duplicate publication, and excluded the protocol, leaving 19 articles (Figure 1).

The 19 articles underwent a quality assessment. All were included in the final analysis.

### 3.1 Quality of the Included Studies

**FIGURE 2:**
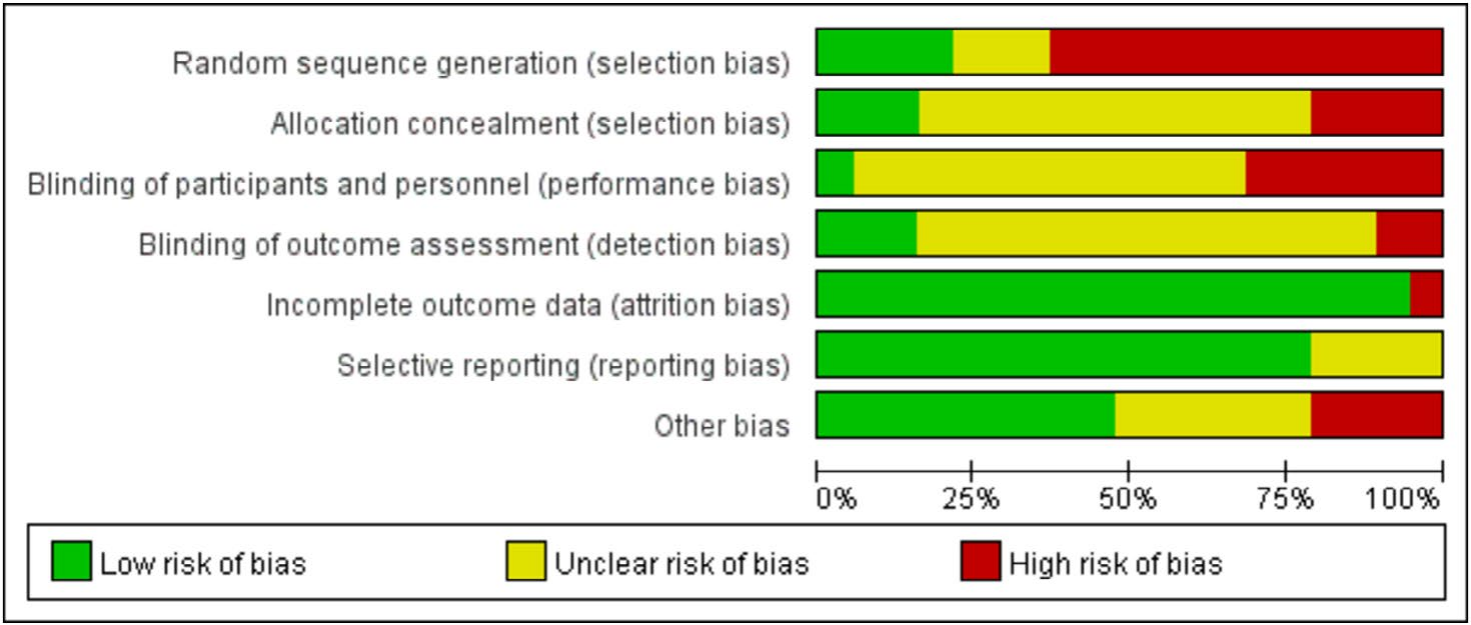
Summary of the risk of bias of the selected studies as assessed by the Cochrane Risk of Bias Assessment tool (2019).

**FIGURE 3:**
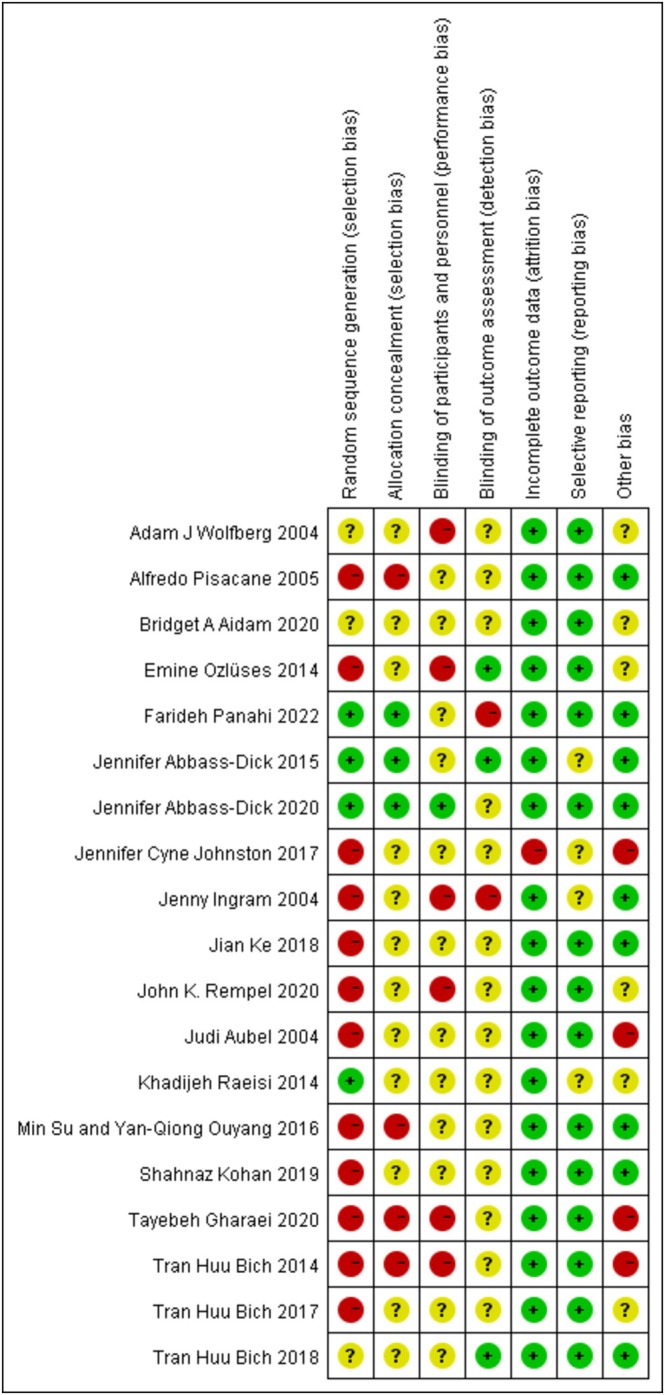
Risk of bias assessed by the Cochrane Risk of Bias Assessment Tool (2019) on each of the included studies.

According to the RoB 2 assessment, the risk of bias was ‘high’ in 14 studies and ‘unclear’ in five (Figure 2). Among these, 12 had a ‘high risk’ of bias in the domain of randomisation (Aubel et al. 2004; Bich et al. 2014, 2018; Bich and Cuong 2017; Gharaei et al. 2020; Ingram and Johnson 2004; Johnston et al. 2017; Ke et al. 2018; Kohan et al. 2019; Ozlüses and Celebioglu 2014; Pisacane et al. 2005; Su and Ouyang 2016), due to the lack of rigorous random sequence generation during allocation concealment, instead using deterministic allocation rules such as alternating or admission dates for the group assignment. The risk of bias was ‘unclear’ in most of the included studies in terms of the blinding of participants and personnel (*n* = 12), and the blinding of outcome assessors (*n* = 14), as none explicitly stated whether blinding was used. In most of the included studies, the risk of bias was ‘low’ for missing outcome data, as all but one reported numbers and reasons for missing data. The exception was Johnston's study (Johnston et al. 2017), which did not specify the reason for missing data on 6‐month exclusive breastfeeding rates in the control group (Figure 3).

### 3.2 Characteristics of the Included Studies

The 19 studies were conducted in the following nine countries: Vietnam (*n* = 4), Iran (*n* = 4), Canada (*n* = 3), Italy (*n* = 2), China (*n* = 2), the Republic of Sierra Leone (*n* = 1), the United Kingdom (*n* = 1), the United States (*n* = 1), and Turkey (*n* = 1).

Only five studies were published before 2014 (*n* = 5), with the remaining 14 published within the last decade (*n* = 14).

The included studies represented diverse healthcare and sociocultural contexts. Eleven studies were conducted primarily in hospital or healthcare settings (*n* = 11), seven in community settings (*n* = 7), and one through a combined community and online platform (*n* = 1). Six studies were implemented in Baby‐Friendly Hospital Initiative (BFHI) settings (*n* = 6), and nine included community support components (*n* = 9). Detailed study characteristics are presented in Table 1, while contextual breastfeeding support environments are presented in Table S1.

**TABLE 1:** Demographics of the participants included in the study (*n* = 19).

Authors, year	Study			Participants			
	Country	Aims of the study	Study settings	Participants	Parity	Age (years)	Sample size
Pisacane et al. 2005	Italy	To investigate whether supporting fathers in recognising their important role in the success of breastfeeding and teaching them how to prevent and manage the most common breastfeeding problems will result in more women breastfeeding	At a university obstetric department in Naples, Italy	Inclusion criteria: parents raising healthy full‐term normal birth weight infants. Exclusion criteria: (1) unmarried women; (2) decision to bottle feed; (3) infants admitted to the NICU	First‐time or experienced parents	No special restrictions	280 (Intervention group: 140 parents vs. Control group: 140 parents)
Ingram and Johnson 2004	England	To assess fathers' and grandmothers' knowledge of breast‐feeding and their ability to support successful breastfeeding. And to design a suitable intervention for fathers and grandmothers to support breastfeeding mothers in an area of relative deprivation, to assess the acceptability and feasibility of the intervention and also monitor its likely effects on breastfeeding rates	At community health centre and family homes in an area of relative social and economic deprivation in South Bristol, UK	Inclusion criteria: women who had expressed an intention to breastfeed or who were undecided	First‐time or experienced healthy mothers' spouses and grandmothers	≥ 18	29 (Intervention group: 29 families vs. Control group: 82 mothers)
Raeisi et al. 2014	Iran	To evaluate the influence of fathers' participation in constant breastfeeding	At a large teaching hospital in Tehran, Iran	Inclusion criteria: (1) healthy mothers with no underlying disease; (2) healthy mothers with no pregnancy complication; (3) being in the second trimester of pregnancy	First‐time or experienced healthy mothers' spouses	≥ 18	100 (Intervention group: 50 fathers vs. Control group: 50 fathers)
Aidam et al. 2020	The Republic of Sierra Leone	To explore how an innovative grandmother‐inclusive approach can be used to address suboptimal infant and young child feeding practices	In two communities: the Torma section of the Bum Chiefdom and the Fikie section	Inclusion criteria: (1) women aged ≥ 17 years; (2) with a child under 2 years old; (3) grandmothers residing with eligible mothers and women aged > 45 years; (4) Living in the community	Grandmothers in the families of first‐time or experienced mothers	≥ 17	147 (Intervention group: 97 grandmothers vs. Control group: 50 grandmothers)
Bich and Cuong 2017	Vietnam	To test the hypotheses of positive changes in the knowledge, attitude, and involvement of fathers in supporting exclusive breastfeeding after receiving breastfeeding education materials and counselling services	At local health centres in two different districts in Vietnam (Chi Linh District, which served as the intervention site, and Thanh Ha District, which served as the control site, both in Hai Duong Province in northern Vietnam)	Inclusion criteria: 7–30 weeks of pregnancy. Exclusion criteria: suffered from serious illness, experience of mental or psychological disorders, miscarriage, refusal to cooperate, or leaving the study site	First‐time or experienced fathers	Not mentioned	469 (Intervention group: 239 parents vs. Control group: 230 parents)
Bich et al. 2018	Vietnam	To examine whether health education to promote fathers' involvement in supporting women is associated with early initiation and exclusive breastfeeding practices	At 13 commune health centres, 1 district hospital and 1 district prevention health centre	Inclusion criteria: (1) mothers and their partners who were 12–27 weeks pregnant; (2) co‐habiting or maintaining regular contact with their wives. Exclusion criteria: (1) women who were wrongly identified as pregnant or had experienced a pregnancy loss (miscarriage, stillbirth, neonatal death) during the follow‐up period; (2) mothers with serious medical problems and unable to answer questions included in the study survey instruments; (3) divorced or separated or migrated out of the study area during the intervention and assessment periods	First‐time or experienced healthy parents	Not mentioned	802 couples (Intervention group: 390 couples vs. Control group: 412 couples)
Abbass‐Dick et al. 2015	Canada	To evaluate the effectiveness of a coparenting intervention on exclusive breastfeeding among primiparous mothers and fathers	At a large teaching hospital in Toronto, Canada	Inclusion criteria: (1) first 2 days postpartum; (2) Singleton birth; (3) 18 years old; (4) 37 weeks gestation at delivery; (5) able to speak and read English; (6) living with a male partner. Exclusion criteria: (1) shared a hospital room with a current study participant; (2) had a medical problem that could interfere with breastfeeding; (3) had an infant who would not be discharged from the hospital with them; (4) did not have access to the Internet or a telephone; (5) planning to breastfeed< 12 weeks; (6) Partner who would not be available to participate in the study	First‐time mothers	≥ 18	214 couples (Intervention group: 107 couples vs. Control group: 107 couples)
Wolfberg et al. 2004	America	To advocate for breastfeeding and to assist fathers if mothers choose to breastfeed	At a large teaching hospital in the United States	Inclusion criteria: (1) women seeking prenatal care; (2) informed consent of their partners	First‐time or experienced expectant fathers	Not mentioned	59 (Intervention group: 27 fathers vs. Control group: 32 fathers)
Kohan et al. 2019	Iran	To design and evaluate whether the empowering programme is beneficial for breastfeeding	At a health centre and hospital in Iran	Inclusion criteria: (1) primiparous and singleton pregnancy; (2) aged over 18 years; (3) low‐risk pregnancy; (4) 32–36 weeks of gestational age; (5) ability to complete the questionnaires; (6) willingness to participate in the study and sharing experiences. Exclusion criteria: participants who were unwilling to continue participating at any stage of the study	First‐time mothers with their main family members	≥ 18	70 (Intervention group: 35 families vs. Control group: 35 families)
Su and Ouyang 2016	China	To evaluate the effectiveness of an educational intervention involving fathers on breastfeeding initiation and exclusive breastfeeding rates, and to explore mothers' perceptions of their partners' support for breastfeeding	At a baby‐friendly university hospital in Wuhan, south‐central China	Inclusion criteria: (1) mothers and fathers aged ≥ 20 years; (2) mothers with gestational weeks of ≥ 39; (3) couples (the mother and the infant's father) who lived together; (4) no gestational complications; (5) first‐time mothers; (6) Singleton foetus; (7) Able to read and understand Mandarin. Exclusion criteria: (1) mothers who experienced serious medical problems or mental illness after delivery; (2) couples who were divorced or planned to migrate to another city during the intervention and follow‐up periods; (3) no access to a telephone; (4) neonatal death; (5) infants with neonatal diseases who were separated from their mothers	First‐time parents	≥ 20	72 (Intervention group: 36 parents vs. Control group: 36 women alone)
Ozlüses and Celebioglu 2014	Turkey	To determine the effect of breastfeeding education provided to the fathers on the rate of exclusive breastfeeding and paternal‐infant attachment	At the Doctor Burhan Nalbantoglu State Hospital Maternity Service in Nicosia	Inclusion criteria: (1) parents able to read, write, and speak Turkish; (2) living in the Turkish Republic of North Cyprus (TRNC) until their infants were 6 months old; (3) with infants having no health problems preventing the early initiation of breastfeeding. Exclusion criteria: (1) parents who might possibly leave the TRNC during the study; (2) who changed health care institutions for postnatal check‐ups; (3) with infants who were not able to breastfeed at an early stage	First‐time or experienced parents	Not mentioned	117 (No intervention group: 39 parents vs. First intervention group: 39 mothers vs. Second intervention group: 39 parents)
Panahi et al. 2022	Iran	To assess the effectiveness of a fathers' educational programme on their support for breastfeeding, mothers' breastfeeding practice, and exclusive breastfeeding status	At a health centre in Karaj‐Iran	Inclusion criteria: (1) men with a primiparous wife; (2) with a healthy single neonate; (3) lacking known medical conditions and/or mental disorders (as stated by the participant); (4) able to Persian. Exclusion criteria: (1) hospitalisation of the neonate; (2) posttraumatic stress disorder associated with the unexpected death of a loved one or couple's separation during the study period; (3) taking medicine that prevents breastfeeding; (4) infant using a pacifier; (5) occurrence of unwanted pregnancy during the study period	First‐time fathers	Mean ± SD for mothers: 22.26 ± 6.63. Mean ± SD for fathers: 29.36 ± 8.01	76 (Intervention group: 38 fathers vs. Control group: 38 fathers)
Ke et al. 2018	China	To explore the influence of a family‐centred breastfeeding education programme in promoting exclusive breastfeeding up to 6 months postpartum and to improve women's attitudes and knowledge, family members' knowledge, and family support	At a prenatal outpatient department of a tertiary hospital in Wuhan, central China	Inclusion criteria: (1) pregnant women aged 20 years or older; (2) 32–34 weeks' gestation; (3) primigravida with single foetus; (4) available for a 6‐month postpartum period following delivery; (5) able to read and understand Mandarin; (6) resides with important family members (infant's father and grandmother/grandmother‐in‐law). Exclusion criteria: (1) women with severe physical diseases (hepatitis B virus, active tuberculosis, human immunodeficiency virus, mammary defect/deformity) and/or a mental illness; (2) suspected foetal congenital malformations (Trisomy 21, cleft lip/palate, hydrocephalus, or heart disease)	First‐time mothers with their main family members (infant's father and grandmother/grandmother‐in‐law)	≥ 20	59 (Intervention group: 29 families vs. Control group: 30 families)
Bich et al. 2014	Vietnam	To determine the extent of exclusive breastfeeding practices among mothers of infants 4 and 6 months old whose fathers received breastfeeding education materials and counselling services	At a community centre integrated with routine health care services for women and children provided by local health staff within the Chi Linh district health system service area	Inclusion criteria: (1) fathers and their pregnant wives whose pregnancies were from 7 to 30 weeks; (2) men who had to live with their wives at home or maintain regular communication with them. Exclusion criteria: (1) women who were wrongly identified as pregnant or had experienced a pregnancy loss (miscarriage, still birth, neonatal death) during the follow‐up period; (2) mothers who had serious medical problems and were unable to answer questions included in the study survey instruments; (3) couples who were divorced or separated or had migrated out of the study area during the intervention and assessment periods (at the pre‐ and post‐tests)	First‐time or experienced fathers	Not mentioned	492 (Intervention group: 251 couples vs. Control group: 241 couples)
Rempel et al. 2020	Vietnam	To examine the effect of the fathering intervention on the parental relationship, exclusive breastfeeding, and infant development	At a father‐centred, community‐based programme integrated into the local health‐care system, with interventions mainly in community health centres	Inclusion criteria: (1) women between 12 and 27 weeks pregnant; (2) couples and newborns who were healthy and free from specific diseases	First‐time or experienced fathers	Not mentioned	733 (Intervention group: 350 couples vs. Control group: 383 couples)
Johnston et al. 2017	Canada	To pilot a group health service delivery model, Centring Parenting, for new parents and assess its feasibility and impact on maternal and infant outcomes	At two Calgary‐area clinics. One clinic is located in an urban setting, not as affluent as the rest of Calgary, and the other clinic is located in a suburban/rural community	Inclusion criteria: (1) full‐term newborns (≥ 37 weeks delivery); (2) first‐time parents with full‐term newborns who received post‐partum visits from PHN in two Calgary‐area Public Health clinics; (3) none of the participants had previously participated in Centring Pregnancy for their prenatal care	First‐time parents	≥ 19	24 (Intervention group: 24 parents)
Aubel et al. 2004	Italy	To strengthen the role of grandmothers in promoting improved maternal and child nutrition practices related to exclusive breastfeeding, improved diet, and decreased workloads during pregnancy	In two health districts, Thiadaye and Joal	Inclusion criteria: (1) grandmothers: people in the family and community who are recognised as having a significant influence on the health and nutrition of mothers and children; (2) young mothers and women of reproductive age (WRA): young mothers and women of reproductive age who receive advice and influence primarily from their grandmothers	Grandmothers in families of women of reproductive age	Not mentioned	200 (Intervention group: 100 grandmothers vs. Control group: 100 grandmothers)
Abbass‐Dick, Newport, et al. 2020	Canada	To compare the effectiveness of eHealth resources and generally available resources, which differed in how breastfeeding information was accessed by mothers and coparents	Recruited in health care providers' offices and services for expectant parents in Ontario and via social media throughout Canada	Inclusion criteria: (1) primiparous or had not previously breastfed; (2) prenatal, after 25 weeks gestation at recruitment; (3) having a singleton birth; (4) at least 18 years old; (5) able to speak and read English; (6) planning to breastfeed; (7) living with a co‐parent. Exclusion criteria: (1) did not have access to the internet or a telephone; (2) were not planning to breastfeed; (3) did not have a co‐parent	First‐time parents	≥ 18	217 (Intervention group: 106 couples vs. Control group: 111 couples)
Gharaei et al. 2020	Iran	To determine the effect of breastfeeding education sessions for primiparous women, with and without the presence of maternal grandmothers, on breastfeeding self‐efficacy and infant feeding patterns	At the Antenatal Clinic of Amiralmomenin Hospital, Tehran, Iran	Inclusion criteria: (1) mothers from 18 to 35 years; (2) having a mother (maternal grandmother); (3) primiparity; (4) low‐risk pregnancy; (5) 31–34 weeks of gestational age; (6) delivery of the infant at Amiralmomenin Hospital. Exclusion criteria: contraindications to breastfeeding that included use of chemotherapy drugs, ergotamines, herpes lesions on the breast, AIDS, and severe depression during pregnancy	First‐time mothers with grandmothers	1835	64 (Intervention group: 32 pairs of mothers and grandmothers vs. Control group: 32 mothers)

### 3.3 Study Population

Among the 19 studies, seven targeted both parents, six focused on fathers only, and four involved grandmothers. Of the grandmother‐focused studies, two included grandmothers alone, one included mothers and grandmothers, and one involved fathers and grandmothers. Two additional studies targeted mothers and their primary family members without specifying the family member type.

In the seven studies targeting both parents (Abbass‐Dick, Sun, et al. 2020; Bich et al. 2018; Johnston et al. 2017; Ozlüses and Celebioglu 2014; Pisacane et al. 2005; Su and Ouyang 2016), sample sizes ranged from 24 to 802 dyads (mean = 246.57, SD = 241.53, median = 214). The six father‐only studies (Bich et al. 2014; Bich and Cuong 2017; Panahi et al. 2022; Raeisi et al. 2014; Rempel et al. 2020; Wolfberg et al. 2004), included 59 to 733 participants (mean = 316.50, SD = 262.64, median = 284.50). Among the grandmother‐involved studies (Aidam et al. 2020; Aubel et al. 2004; Gharaei et al. 2020; Ingram and Johnson 2004; Ke et al. 2018; Kohan et al. 2019), two grandmother‐only studies included 147 and 200 participants, while the remaining four ranged from 29 to 70 participants (Aidam et al. 2020; Aubel et al. 2004).

### 3.4 Theoretical Framework of the Interventions

Among the 19 studies, only five utilised a theoretical framework in their intervention design. Of these, one (Bich et al. 2018) was based on Albert Bandura's Social Cognitive Theory and Icek Ajzen's Theory of Planned Behaviour (Bandura 2004; Ajzen 1991); one (Abbass‐Dick et al. 2015) used the co‐parenting theory proposed by Feinberg (Feinberg 2003); one (Kohan et al. 2019) used the Empowerment Theory proposed by Barbara Solomon in 1976 (Kabeer 2005); one (Rempel et al. 2020) used the Ecological Theory and Breastfeeding Team Theory proposed by Parke and Doherty (Rempel et al. 2017); and one (Aubel et al. 2004) was based on a systems framework. This study also introduced the health‐seeking paradigm, Kleinman's concept of a three‐sector health system (biomedical, traditional and family sectors), and the notion of family health production as the theoretical foundation (Chrisman 1977).

### 3.5 Intervention Content

Of the 19 studies, the main intervention components focused on: (1) breastfeeding knowledge and benefits (*n* = 10), including the importance of exclusive breastfeeding and the composition and benefits of breast milk; (2) breastfeeding skills and management (*n* = 8), such as breastfeeding positions and breast milk storage; (3) management of breastfeeding problems (*n* = 7), including prevention and treatment of common conditions such as engorgement, mastitis, nipple pain, and infant refusal; (4) father and family support (*n* = 9), aimed at promoting fathers' roles and family involvement in breastfeeding; (5) parenting knowledge (*n* = 5), including infant care and recognition of feeding cues; and (6) psychological support and education (*n* = 5), focusing on improving breastfeeding self‐efficacy and providing emotional and practical support. The detailed intervention characteristics are summarised in Table 2.

**TABLE 2:** Intervention strategies targeting breastfeeding (*n* = 19).

Authors, year	Theoretical foundation and components of intervention	Delivery format	Onset of intervention	Dosage, frequency, duration	Facilitator	Fidelity	Data collection	Main results	Main findings
Pisacane et al. 2005	No specific theory. (1) infant feeding and the difficulties associated with breastfeeding; (2) transitional breastfeeding crises; (3) return to outside employment; (4) breast swelling, mastitis, sore and inverted nipples, refusal to breastfeed, and unsure how to deal with; (5) help fathers recognise and accept their role in breastfeeding success	(1) Breastfeeding leaflet; (2) face‐to‐face conversation	Postpartum	(1) Breastfeeding leaflet: the day after delivery, once, parents were provided with a leaflet on the benefits and management of breastfeeding and an explanation of the leaflet; (2) face‐to‐face conversation: 40 min, once, with the intervention fathers and a leaflet distributed at the end of the conversation	Midwife	The midwife who conducted the intervention was trained through the WHO‐UNICEF 40‐h training course	(1) Baseline: second day postpartum; (2) breastfeeding status: 6 months postpartum, 12 months postpartum, by telephone; (3) difficulties in breastfeeding: when mothers perceived that their milk was insufficient, and mothers who stopped breastfeeding due to difficulties and problems; (4) breastfeeding help received from partners; (5) breastfeeding incidence: 12 months postpartum	(1) The rate of exclusive breastfeeding at 6 and 12 months postpartum was significantly higher in the intervention group; (2) mothers in the control group felt that they had insufficient milk and abandoned breastfeeding due to breastfeeding problems significantly more frequently than mothers in the intervention group; (3) the intervention group reported receiving support and relevant help from their partners in infant feeding	(1) Fathers play an important role in supporting successful breastfeeding and increasing breastfeeding rates, and mothers' decisions about how to feed their babies; (2) supporting fathers during breastfeeding may help to increase mothers' satisfaction with breastfeeding, the duration of breastfeeding, and both parents' adjustment to parenting
Ingram & Johnson, 2004	No specific theory. (1) The health benefits of breastfeeding; (2) breastfeeding positions; (3) breastfeeding management; (4) how families can support breastfeeding	By means of leaflets: fathers and grandmothers were each given the same breastfeeding leaflet	Prenatal: around 36 weeks	(1) Focus groups and interviews: prenatal, once; (2) intervention with partners and grandmothers at the mothers' home: at approximately 36 weeks prenatally, about 30 min, once; (3) an interview: in the mother's home at about 8 weeks postpartum, about 30 min, once	Research midwife	Not mentioned	(1) Baseline: approximately 36 weeks prenatal; (2) post‐intervention: 8 weeks postpartum	(1) Mothers in the intervention group had a higher breastfeeding rate at 8 weeks postpartum than other mothers who did not receive the intervention; (2) mothers who were still breastfeeding at 8 weeks postpartum received more practical and emotional support in the postpartum period than bottle‐feeding mothers	Fathers and grandmothers have an important role to play in providing emotional and practical support to breastfeeding mothers
Raeisi et al. 2014	No specific theory. (1) Benefits of breastfeeding; (2) contraindications of breastfeeding; (3) milk storage; (4) understanding the signs of an infant's hunger and satiety	By breastfeeding training sessions at the Family Health Research Center	Prenatal: 30 weeks to the end of pregnancy	Breastfeeding training courses: 3 times, from the 30th week of pregnancy to the time of delivery	Not specifically mentioned	Not mentioned	(1) Baseline: 30 weeks of gestation; (2) post‐intervention: 1 month, 3 months, 6 months postpartum	(1) Fathers in the intervention group had a higher level of knowledge about breastfeeding than those in the control group; (2) the likelihood of breastfeeding in the families of fathers in the intervention group was about 6 times greater than in the control group; (3) fathers in the intervention group were 11 times more likely to participate in, encourage, and support breastfeeding than those in the control group	The involvement of fathers had a positive impact on improving their knowledge and attitudes towards breastfeeding, and they supported their spouses more
Aidam et al. 2020	No specific theory. (1) Affirm the role of grandmothers and increase their openness to new ideas; (2) disseminate information about knowledge that promotes child growth and development, healthy behaviours, nutrition, and early childhood development	(1) “Grandmother Praise Day”; (2) group meetings; (3) intergenerational forums; (4) antenatal and postnatal care visits; (5) Community Health Worker Home Visits	Postpartum	(1) ‘Grandmother Praise Day’: unscheduled, throughout the intervention period; (2) group meetings: once a month, throughout the intervention period; (3) intergenerational forums: once a month, throughout the intervention period; (4) antenatal and postnatal care visits: standard care is provided by the Ministry of Health through antenatal and postnatal care visits, the exact frequency of which is not clear; (5) Community Health Worker Home Visits: standardised care is provided by Community Health Workers based on training by World Vision staff, the exact frequency of which is not clear	(1) World Vision staff; (2) an adult education expert; (3) grandmother leaders; (4) community health workers	(1) Grandmother leaders were selected by their communities based on community defined criteria (well respected, experience caring for pregnant women and mothers with young children, and/or traditional birth attendants); (2) grandmother leaders from all communities attended quarterly stakeholder meetings where their leadership abilities, facilitation, and their understanding of optimal MCHN practices skills were strengthened; (3) community health workers were trained by World Vision staff	(1) Baseline survey: 60–90 min; (2) endpoint cross‐sectional survey: 60–90 min	(1) Grandmothers in the intervention group had higher levels of overall nutritional knowledge than those in the control group; (2) the attitudes and beliefs of intervention grandmothers improved from baseline to endline, whereas those of control group grandmothers did not; (3) there was no difference in early breastfeeding rates between the intervention and comparison groups at the endline; (4) a higher proportion of children in the intervention group were exclusively breastfed in the first week of life, but there was no significant difference in the proportion exclusively breastfed among 0–6‐month‐olds, nor in the proportion continuing to breastfeed among 6–23‐month‐olds; (5) in terms of complementary feeding, a significantly higher proportion of children aged 6–23 months in the intervention group achieved minimum dietary diversity and a minimum acceptable diet	A grandmother‐ inclusive approach has the potential to improve grandmothers' and mothers' knowledge, attitudes, and infant and young child feeding practices
Bich and Cuong 2017	No specific theory. (1) Knowledge and skills of breastfeeding; (2) implementation and mobilisation of the father's role	(1) Mass media dissemination; (2) individual counselling; (3) group counselling; (4) home visits; (5) conducting public activities	Prenatal: 7–30 weeks	(1) Mass media dissemination: broadcasting over the loudspeaker system in the local community twice a week; (2) group counselling: offered on the 25th of every month; (3) individual counselling and home visits: 4 sessions, including one and three during the prenatal and postnatal period, respectively; (5) conducting public activities: lasted for 1 year	Local health staffs within the Chi Linh District health system periphery and trained village health workers	Not mentioned	(1) Baseline: 7–30 weeks before delivery; (2) structured interviews: postpartum; (3) knowledge, attitude and practice of the fathers regarding breastfeeding: fathers of 2.5 to 4‐month‐old infants	(1) Fathers' knowledge of breastfeeding and their understanding of its importance was significantly higher in the intervention group than in the control group; (2) the proportion of those who breastfed for 6 months was significantly higher in the intervention group than in the control group; (3) fathers in the intervention group had more favourable attitudes towards exclusive breastfeeding and early initiation of breastfeeding, and rates of breastfeeding for infants at 6 months than fathers in the control group; (4) the proportion of fathers involved in caring for the mothers was significantly higher in the intervention group than in the control group; (5) a higher proportion of fathers in the intervention group than in the control group asked the mothers to breastfeed regularly after childbirth and participated in various forms of breastfeeding support; (6) fathers in the intervention sites were twice as likely as fathers in the control sites to be involved in supporting exclusive breastfeeding at the health facility immediately after delivery, and to inform mothers and family members about best breastfeeding practices and to take care of the mother's health and nutrition; (7) fathers' improved breastfeeding knowledge provided a strong foundation for a change in their attitude and engagement in support of breastfeeding during the first 6 months	The intervention was associated with improvements in fathers' breastfeeding knowledge, attitudes, and practices
Bich et al. 2018	Social cognitive theory. Planned behaviour theory. (1) Dissemination of information on breastfeeding; (2) Share breastfeeding experiences and encourage others to breastfeed	(1) Mass media communication; (2) paternal group training and individual counselling at home visit; (3) fathers' clubs; (4) Fathers' group on Facebook	Prenatal: ≥ 28 weeks	(1) Mass media communication: twice a week for a total of 132 sessions; (2) paternal group training: group counselling: 1 time per month, 30–45 min/time, 110 times in total; a total of 190 people received counselling at 3.5 months postpartum; (3) individual counselling at home visits: 4 times in total, the first time in the last 3 months of the prenatal period, the second time in the first month of the postnatal period, the third time in the 6 weeks of the postnatal period, and the fourth time in the 3.5 months of the postnatal period, for a total of 432 visits; (4) fathers' clubs: divided into monthly meetings and ad hoc meetings, lasting 3 months, with about 360 participants; (5) organisation of fathers' group on Facebook: continued after the 3 months of fathers' clubs	10 district hospital midwives, 26 commune health workers and midwives with a 2‐day workshop training	Based on the findings of an equivalent study	(1) Baseline: 12–27 weeks of gestation; (2) post‐intervention: 1, 4, and 6 months postpartum	(1) Mothers in the intervention group initiated breastfeeding within 1 h after delivery, and the rates of breastfeeding were higher than those of the control group at 1, 4, and 6 months postpartum; (2) the incidence of cessation of breastfeeding in the intervention group was lower than the risk in the control group at 1, 4, and 6 months postpartum; (3) the intervention group was 1.7 times more likely to breastfeed early than mothers in the control group; (4) the intervention group was 10 times and 7.5 times more likely to breastfeed at 1 and 4 months postpartum than mothers in the control group	Intervention to involve fathers in breastfeeding promotion has added additional positive evidence of the effects of the intervention on early initiation of breastfeeding and exclusive breastfeeding rates at 1, 4, and 6 months
Abbass‐Dick et al. 2015	Coparenting theory. (1) The role of breastfeeding; (2) how fathers should help with breastfeeding; (2) extensive information on breastfeeding and co‐parenting	(1) In‐hospital discussion; (2) breastfeeding booklet and co‐parenting booklet; (3) video, website, e‐mail, telephone call	Postpartum: 2nd day postpartum	(1) In‐hospital discussion: a 15 min face‐to‐face discussion with a breastfeeding specialist; (2) breastfeeding booklet, co‐parenting booklet, website; (3) video: an 11‐min video on breastfeeding and parenting education; (4) e‐mail: at 1 week and 3 weeks after delivery, e‐mails were sent to parents to assist the couples in navigating through the intervention information package and serve as a reminder of the resources provided; (5) telephone call: at 2 weeks postpartum	Lactation Specialist	Check on the use of breastfeeding and co‐parenting resources by e‐mail and phone	(1) Baseline: 2 days postpartum; (2) post‐intervention: 6 weeks postpartum, 12 weeks postpartum	(1) The number of mothers breastfeeding in the intervention group was higher than those in the control group at 6 weeks postpartum and 12 weeks postpartum (statistically different at 12 weeks postpartum); (2) mothers' perceptions of the parent–child relationship, mothers' perceptions of support, fathers' self‐efficacy in breastfeeding, and fathers' attitudes towards infant feeding; BSES‐SF scores were higher in the intervention group than in the control group at 6 weeks postpartum and 12 weeks postpartum; (3) there was a significant increase in the number of mothers receiving help from their partners in the first 6 weeks postpartum in the intervention group compared to the control group; (4) mothers in the intervention group were significantly more satisfied with their partner's involvement in breastfeeding and with the breastfeeding information they received than those in the control group	Co‐parenting and breastfeeding interventions promote improvements in breastfeeding duration, fathers' breastfeeding self‐efficacy, and mothers' perceptions of fathers' involvement and assistance in breastfeeding
Wolfberg et al. 2004	No specific theory. (1) Infant care; (2) breastfeeding promotion	(1) Face‐to‐face group course discussions; (2) video; (3) role‐playing; (4) facilitators explaining core information	Prenatal	Face‐to‐face group discussions: 2 h once every 2 weeks, in groups of 4–12 fathers	An easygoing and engaging, knowledgeable without being overbearing classroom facilitator who was black and was a father himself	By the end of the class, the participants were able to explain the benefits of breastfeeding and to facilitator	(1) Baseline: when attending the programme prenatally; (2) post‐intervention: data on fathers were collected after each intervention; data on mothers were collected at 2 weeks postpartum, 4 weeks postpartum, and 8 weeks postpartum	(1) Mothers whose partners attended breastfeeding classes were more likely to initiate breastfeeding; (2) mothers' breastfeeding intentions in the first month of life, the beliefs of the infant's maternal grandmother that the infant should be breastfed, and the beliefs of the infant's father that the infant should be breastfed were all associated with an increased incidence of breastfeeding	Expectant fathers can be influential advocates for breastfeeding, playing a critical role in encouraging a woman to breastfeed her newborn infant
Kohan et al. 2019	Empowerment theory. (1) The benefits of breastfeeding; (2) appropriate techniques for breastfeeding; (3) prevention and treatment of problems in breastfeeding; (4) mother's milk expression and storage; (5) increasing mother's milk and nutrition during pregnancy and postpartum period; (6) caring for the child; (7) family's collaboration and support for breastfeeding	(1) Small‐group breastfeeding education; (2) lectures, discussions, simulations, teaching software and pamphlets; (3) face‐to‐face breastfeeding counselling; (4) telephone counselling	Prenatal: 32–36 weeks; Postnatal: 3–5 days after delivery and when experiencing breastfeeding problems	(1) Small‐group breastfeeding education: two 2‐h group breastfeeding education sessions; (2) lectures, discussions, simulations, teaching software and pamphlets; (3) face‐to‐face breastfeeding counselling: one breastfeeding counselling session for mothers, their spouses and their family members, 3–5 days after the mother's delivery; (4) telephone counselling: at any time interventionists were needed	A researcher	The researcher was certified for lactation consultation and was responsible for the education and counselling sessions	(1) Basic questionnaire; (2) women's breastfeeding empowerment: at 2 weeks, 2 months, and 4 months postpartum	(1) The total mean breastfeeding empowerment score of the intervention group was significantly higher than that of the control group at 2 weeks, 2 months and 4 months after delivery; (3) the mean scores of the intervention group in the seven domains of breastfeeding knowledge, attitudes and skills, skills in preventing and resolving breastfeeding problems, breastfeeding adequacy, negotiating and obtaining family support, and breastfeeding self‐efficacy were significantly higher than those of the control group at 2 weeks postpartum and 2 months postpartum compared to baseline; (4) exclusive breastfeeding rates in the intervention group at 2 weeks postpartum and 2 months postpartum were significantly higher than in the control group; (5) breastfeeding family support and family participation were higher in the intervention group than in the control group	Breastfeeding empowerment education enhances the knowledge and beliefs about breastfeeding of mothers and their main family members, increases family involvement and family support for breastfeeding, and therefore should be developed and established during pregnancy and 2 weeks after delivery
Su and Ouyang 2016	No specific theory. (1) Basic knowledge of breastfeeding: breastfeeding techniques; (2) involvement in decision making on feeding model, emotional and practical support for breastfeeding; (3) correct breastfeeding positions, practice; (4) demonstrate how fathers can help during breastfeeding	By face‐to‐face offline breastfeeding education programme courses	Prenatal: ≥ 39 weeks	Face‐to‐face offline breastfeeding education programme courses: 60–90 min, 4–8 participants in each class	Researchers	The educational materials were selected from the websites of the World Health Organization and La Leche League International. The educational intervention was tested by three obstetricians, three senior midwives, and three senior nurses working in an obstetrics unit	(1) Baseline: baseline questionnaire, before the intervention, included parental age, income, education level, sex and weight of newborns, and the mode of delivery; (2) breastfeeding attitude: The Iowa Infant Feeding Attitude Scale (IIFAS), before and after the intervention; (3) breastfeeding knowledge level: Breastfeeding Knowledge Scale (BKS), before and after the intervention; (4) face‐to‐face interview: 4 times after delivery; the first interview was upon hospital discharge. The second to fourth times were at 1, 4, and 6 months postpartum and conducted by telephone, and included Infant feeding patterns (exclusive breastfeeding versus complementary feeding), father's attitude towards breastfeeding and how they support breastfeeding	(1) There was a significant increase in the breastfeeding attitudes and breastfeeding knowledge of both mothers and fathers after the intervention, especially in the breastfeeding knowledge scores of the fathers; (2) the rates of exclusive breastfeeding at 1 month postpartum were similar in the intervention and control groups, but fewer mothers in the intervention group chose formula feeding than those in the control group; exclusive breastfeeding rates in the intervention group were significantly higher at 4 and 6 months postpartum than those in the control group; (3) the formula feeding rate at 6 months of age was significantly lower in the intervention group than in the control group; (4) most of the mothers in both the intervention and control groups claimed that their partner's attitude towards breastfeeding was positive and supportive in their talk about partner support after delivery, but mothers in the intervention group said that their partners knew how to support breastfeeding and took care of their babies and did some household chores with a positive attitude, and that when mothers faced challenges related to breastfeeding, they received firm support from their partners to continue breastfeeding; Mothers in the control group reported that their partners wanted to help but did not know how	Fathers may positively influence the breastfeeding practices of mothers; they can be mobilised in programmes aimed at improving the early initiation of breastfeeding
Ozlüses and Celebioglu 2014	No specific theory. How to breastfeed	By face‐to‐face offline breastfeeding education	Postpartum: from the day the mother came for delivery	Face‐to‐face offline breastfeeding education: 20 min/day for each parent, from the day the mother came for delivery and continued to discharge	Researchers	Not mentioned	(1) Baseline: measured before delivery; (2) exclusive breastfeeding rate: 1, 2, 4, and 6 months postpartum; (3) parent–child attachment relationship	(1) From the 1st month to the 6th month, the breastfeeding rate of the intervention groups of both mothers and fathers who had received EBF education was higher than that of the control group; (2) the mean PIAS score of the intervention groups of both mothers and fathers who had received EBF education was higher than that of the other groups and was closest to the top; (3) the fathers of the first experimental group had no EBF education, but their scores were higher than the mean of 79, so the mother's education also had a positive effect on the fathers' scores	Providing breastfeeding education to fathers during the postpartum period significantly increases exclusive breastfeeding rates and strengthens paternal attachment
Panahi et al. 2022	No specific theory. (1) The composition of breast milk; (2) the importance and benefits of exclusive breastfeeding; (3) the correct positions for breastfeeding; (4) fathers' role in promoting and continuing breastfeeding; (5) the ways to encourage mothers or planning for regular exclusive breastfeeding; (6) how to free up adequate time for mothers to rest	By face‐to‐face education and counselling	Postnatal: 2 and 3 weeks after delivery	Face‐to‐face education and counselling: two 40‐min sessions at one‐week intervals, at 2 and 3 weeks after delivery	Researchers who were blinded	Not mentioned	(1) Demographic and Maternal–Infant Information; (2) fathers' support for breastfeeding: pre‐intervention, 4 months postpartum; (3) mothers' breastfeeding practice: pre‐intervention, 4 months postpartum; (4) exclusive breastfeeding status: pre‐intervention, 4 months postpartum	(1) In the intervention group, ‘father's support for breastfeeding’ was 31.93 units, significantly higher than that of the control group after 4 months, and the average score on father's support in the post‐intervention group was 22.15 units higher than that of the pre‐intervention group; (2) mothers' breastfeeding practice scores in the intervention group were 26.32 units, significantly higher than those in the pre‐intervention and control groups, and the mean score of mothers' breastfeeding practice in the post‐intervention period was 12.86 units higher than that in the pre‐intervention period; (3) the frequency of exclusive breastfeeding was higher at 4 months after the intervention	Father's education improves mothers' breastfeeding practice and increases the rate and continuity of exclusive breastfeeding
Ke et al. 2018	No specific theory. (1) Benefits of breastfeeding and how to breastfeed; (2) breastfeeding positions; (3) problems from breastfeeding and solutions; (4) common health problems of newborn babies; (5) storage of breast milk when returning to work; (6) how to choose a breast pump to express milk by hand	(1) Prenatal breastfeeding education lectures; (2) home visits; (3) telephone and SMS video/audio interactions; (4) breastfeeding problem counselling	Prenatal: 34 weeks	(1) Prenatal breastfeeding education lectures: 45 min each time, prenatal, 2 times in total, at 34 weeks and 36 weeks prenatal; (2) home visits: 30 min each time, 1 month postpartum, 3 times in total, on the 7th, 14th, and 28th day postpartum, when the father and grandmother are at home; (3) telephone and SMS video/audio interactions: 15 min each time, 8 times every 2 weeks from 2 months postpartum to 6 months postpartum; (4) counselling on breastfeeding issues: at any time when they encounter breastfeeding problems and have access to information via the internet/public information platform	Researchers	(1) The researcher had completed 90 h of education in human lactation and breastfeeding; (2) the breastfeeding education programme was reviewed by two obstetricians, a paediatrician, an International Board Certified Lactation Consultant and a nurse educator, and the content of the education programme was reached by consensus; (3) the educational and informational support used during the home visits, calls and responses, and WeChat/phone conversations was developed based on the Core Curriculum for Lactation Consultant Practice and Medications and Mothers' Milk	(1) Demographic questionnaire: part 1, administered prenatally, including race, age, mother's education, father's education, and monthly income; part 2, administered postnatally, included mode of delivery, maternity leave, and early separation of mother and baby, collected at 6 months postpartum; was completed during the face‐to‐face interviews; (2) breastfeeding behaviour in the first 6 months: 7 days,1 month, 4 months and 6 months postpartum; (3) feeding attitudes: 7 days, 1 month, 4 months, and 6 months postpartum; (4) perceived family support: 7 days, 1 month, 4 months, and 6 months postpartum; (5) breastfeeding knowledge: 7 days, 1 month, 4 months, and 6 months postpartum	(1) The intervention group was more likely than the control group to exclusively breastfeed their babies in the first 6 months; (2) mean subject knowledge and father knowledge were significantly higher in the intervention group than in the control group at all timepoints; (3) maternal knowledge after prenatal lectures and at 1 month postpartum was significantly higher in the intervention group than in the control group; (4) the mean values of perceived family support at 1 month, 4 months, and 6 months postpartum were significantly higher in the intervention group than in the control group	Family support may be a facilitator of breastfeeding
Bich et al. 2014	No specific theory. (1) The benefits of breastfeeding; (2) the ways in which fathers support their wives to breastfeed	(1) Mass media dissemination; (2) group counselling; (3) individual counselling; (4) telephone consultations; (5) public events	Prenatal: early pregnancy, and lasts from prenatal to postnatal	(1) Mass Media Dissemination: the local public loudspeaker system (broadcasting twice a week); (2) group counselling: 30–45 min per session, 49 group counselling sessions in total, seven in each of the seven commune health centres, with a total of 545 participants; (3) individual counselling: 4 home visits per family (1st home visit in late pregnancy, 2nd home visit in the first week of the postnatal period, 3rd home visit at 42 days into the postnatal period, and 4th home visit at 3.5 months into the postnatal period); a total of 239 eligible fathers received a total of 862 home visits, for an average of 3.6 visits per person; (4) telephone consultations: at any time that the interventionists had problems with breastfeeding; (5) public events: after delivery	Trained village health workers	Not mentioned	(1) Baseline information: included parents' age, education and occupation, place of residence, family size, family structure, and family's economic status, and the father's economic status in the family; (2) breastfeeding rates: at 4 and 6 months postpartum, through a questionnaire modified from tools used in a previous survey measuring child‐feeding practices in Cambodia; the first visit asked about basic infant characteristics, including the infant's sex, birth weight, type of delivery, and birth order; breastfeeding practices at 4 and 6 months postpartum were asked about, including the early initiation of breastfeeding, and other feeding practices at 4 and 6 months	(1) The rate of exclusive breastfeeding was higher in the intervention group than in the control group, especially at 6 months postpartum; (2) mothers in the intervention group were approximately twice as likely as control group mothers to be exclusively breastfeeding at 4 months, and mothers in the intervention group were six times more likely than control group mothers to be exclusively breastfeeding at 6 months	The involvement of fathers in the care continuum, both at health care facilities and in households, might increase the proportion of mothers who adopt exclusive breastfeeding at 4 and 6 months of age
Rempel et al. 2020	Ecological theory. Breastfeeding team theory. (1) Information on the importance of exclusive breastfeeding; (2) the principles of good‐quality father involvement; (3) the importance of using a teamwork approach	(1) Antenatal group meetings; (2) Prenatal home visits and individual postnatal home visits; (3) postnatal visits; (4) community radio; (5) postnatal training; (6) community fathers' clubs	Prenatal	(1) Antenatal group meetings: once; (2) prenatal home visits and postnatal visit: at approximately 1, 6, and 15 weeks postpartum; (4) community radio: 5–10 min, weekly broadcasts; (5) postnatal training: 2 days before birth; (6) community fathers' clubs: approximately 6 months after the club was formed, planned a friendly fathers' competition that took place	One or two physicians or physician's assistants from each of the 13 intervention commune health centres	Physicians or physician's assistants received a 2‐day training programme. Members of the study team responsible for managing the staff checked in regularly with community health workers to discuss how they were managing the group meetings and home visits and to assess the extent to which they were implementing the intervention	(1) Baseline: demographics, family composition, household economic status (HES), and relationship quality data, collected at recruitment; (2) childbirth information: at 1 month postpartum; (3) Partner Breastfeeding Support Behaviours: at 1 and 4 months, Partner Breastfeeding Influence Scale (PBIS), father and mothers independently completed; (4) exclusive breastfeeding duration: at 1, 4 and 6 months postpartum (at 1 and 4 months, by face‐to‐face interviews; at 6 months by telephone interview); (5) parental relationship quality: at baseline and at 4 months postpartum internationally validated 16‐item self‐report measure developed by Gere and MacDonald; (6) infant developmental status: at 9 months of age, Developmental Milestones Checklist–II	(1) Mothers in the intervention group rated the fathers significantly higher than mothers in the control group on helping and responding; (2) all forms of father‐supportive behaviour were associated with longer exclusive breastfeeding; (3) higher levels of partner breastfeeding support, particularly support that is sensitive and responsive to the mother's needs and wants, will be associated with longer exclusive breastfeeding duration; (4) improvements in the quality of the mothers' own relationships were significantly associated with the mothers' experiences of various types of father support; (5) all support from both fathers and mothers significantly predicted improvements in children's language scores; the higher the father's ratings on Helpfulness and Reactivity, the higher the infant's Individual‐Social Score and motor development scores; (6) changes in the quality of the mother's relationship predicted higher language scores	(1) Educating and encouraging fathers to become more involved with mothers and babies as part of the parenting team in multifaceted, community‐based interventions can have a significant impact on the couple's relationship and the baby's life; (2) interventions are effective at changing the amount of support fathers provide to mothers, and in terms of sensitivity and responsiveness to the amount and type of support mothers need; (3) mothers' perceptions of fathers' helpfulness, sensitivity, and responsiveness are associated with longer periods of exclusive breastfeeding, and fathers' responsive behaviours, and predict the quality of the improvement in the relationship and development of the infant
Johnston et al. 2017	No specific theory. (1) Maternal themes: weight goals, role fulfilment, breastfeeding, maternal health, depression, and contraception; (2) infant themes: health, development, and nutrition; (3) other themes: sexuality, mental health, parenting, family relationships, and domestic violence	(1) Family group meetings; (2) integrated health services and assessments	Postpartum: 1 month after the baby's birth	(1) Family group meetings: six sessions in the first year after the birth of a child, each lasting 2 h, at 1, 2, 4, 6, 9, and 12 months postpartum, with three to seven families attending the group together; (2) integrated health services and assessments	Public health nurse	Two public health nurses who had received 2 days of training in the CP model and its implementation	(1) Baseline and demographic data: breastfeeding initiation and duration, postpartum depression, parenting experience, anxiety, social support and stress data, as well as programme satisfaction and infant vaccination data; (2) parental psychosocial well‐being: collected at 1, 2, 4, 6, 9, and 12 months post‐intervention using The Edinburg Postnatal Depression Scale (EPDS), the Parenting Morale Index, the Perceived Stress Scale, the MOS Social Support Survey, the Spielberger State–Trait Anxiety Scale, and the Spielberger State–Trait Anxiety Scale	(1) Mothers in the intervention group were more confident in their parenting skills and more active in their role as parents; (2) mothers in the intervention group reported knowing where to get answers to their parenting questions and managing stress better after the programme. There were no significant differences in mental health between the intervention and conventional groups; (3) about 36% of mothers in the intervention group were exclusively breastfeeding at 4 months postpartum, 80% of mothers in intervention group were still breastfeeding at 6 months postpartum, and 42% of mothers in the intervention group were predominantly formula‐feeding at 12 months postpartum; (4) the vaccination rate of infants in the intervention group was higher than that of the control group at 12 months postpartum	The centre parenting intervention may have the potential to address the needs of many new parents and positively impact the parenting experience
Aubel et al. 2004	Based on a systems framework incorporating precepts from the health‐seeking model, Kleinman's notion of the tri‐sectoral health system (biomedical, traditional and family sectors) and the household production of health. (1) Educate and empower grandmothers. (2) Improve nutritional knowledge and attitudes; and (3) improve young mothers' practices. (4) Enhancing family and community maternal and child health. (5) Praise and teaching songs	(1) Socio‐cultural appreciation activities; (2) new nutrition sessions	Prenatal	(1) Socio‐cultural appreciation activities; (2) New Nutrition Sessions: 4 sessions in each community, conducted in 13 communities over a period of 9 months	A cadre of community animators and CCF/MOH staffs	Community animators have been well‐trained	(1) Individual interviews: before the start of the intervention and 12 months after the intervention; (2) structured questionnaire; (3) focus group interviews: after 1 year of the intervention	(1) The number of grandmothers who advised breastfeeding for 5 months more than doubled; (2) there was a significant increase in the best practice of introducing complementary foods for the first time at 5–6 months (in line with the Department of Health's Early Years Policy); (2) a post‐intervention comparison of the pre‐ and post‐test interview data of grandmothers in the intervention community revealed significant changes in all eight parameters related to their nutrition‐related knowledge and advice; (3) breastfeeding rates in the communities of the intervention group increased significantly compared to the control group	The intervention significantly improved the knowledge and advice of the grandmothers, and changes in their advice led to changes in the practices of women of reproductive age
Abbass‐Dick, Sun, et al. 2020	No specific theory. (1) Why and how to breastfeed; (2) common concerns in the early days; (3) supporting mothers/fathers/partners; (4) where to get help; (5) extensive information on how to work as a team to meet breastfeeding goals	E‐health resources	Prenatal: ≥ 25 weeks	E‐health resources: throughout the perinatal period	Researcher	Researchers sent emails weekly for 6 weeks to both two groups reminding them that they were in the study	(1) Baseline demographic variables; (2) delivery details: at 4 weeks postpartum; (3) exclusive breastfeeding: at 4, 12, and 26 weeks postpartum; (4) breastfeeding duration: at 4, 12, 26, and 52 weeks postpartum; (5) supplementation details: at 4, 12, and 26 weeks; (6) breastfeeding problems; (7) maternal and paternal breastfeeding self‐efficacy: at baseline, 2 weeks post enrolment and 4 weeks postpartum; (8) breastfeeding knowledge: at baseline, 2 weeks post enrolment, and at 4 weeks postpartum; (9) infant feeding attitude: at baseline, 2 weeks post enrolment, and 4 weeks postpartum; (10) breastfeeding partner support; (11) breastfeeding co‐parenting; (12) intervention use; (13) breastfeeding resource use	(1) Mean scores for the secondary outcomes of breastfeeding knowledge, infant feeding attitudes, and breastfeeding self‐efficacy in the intervention group showed modest and statistically significant increases from baseline to 2 weeks after enrolment and 4 weeks postpartum compared with SC2; (2) the breastfeeding partner support scores and co‐parent breastfeeding scores of the intervention group were higher at 12 and 52 weeks postpartum than those of the control group; (3) participants indicated that the top three topics that they learned were: why breastfeeding is important, how to successfully obtain milk, and how to overcome common problems and solutions; the majority of participants indicated that the resource helped them to overcome problems such as babies not suckling, insufficient milk, sore nipples, bleeding, and ruptures	Mothers and their co‐parents should have targeted breastfeeding education and support interventions, and web‐based resources should be designed to meet the needs of families of childbearing age
Gharaei et al. 2020	No specific theory. (1) The importance and benefits of breast milk; (2) the symptoms of hunger and fullness, ways to diagnose the adequacy of milk; (3) breastfeeding positions; (4) common breast problems and their prevention and treatment; (5) ways to increase the amount of milk, complications with the use of a bottle, formula, and pacifier, diseases and breastfeeding, and drug use during breastfeeding; (6) support for the lactating mother	(1) Breastfeeding education; (2) post‐natal meeting	Prenatal: 31–34 weeks	(1) Breastfeeding education: two times, provided at 31–34 weeks of pregnancy and 35–37 weeks of pregnancy; (2) postnatal meeting: approximately 3 h prior to discharge	Researchers	Not mentioned	(1) Basic characteristics: 31–34 weeks of gestation; (2) breastfeeding self‐efficacy: 24 h, 4 and 8 weeks postpartum; (3) infant feeding information: 4 and 8 weeks postpartum	(1) Breastfeeding self‐efficacy scores were significantly higher in the intervention group than in the control group at 4 weeks postpartum and 8 weeks postpartum; (2) breastfeeding rates in the intervention group were higher than those in the control group at 4 weeks postpartum and 8 weeks postpartum, but there was no significant difference	Breastfeeding education involving grandmothers is an effective way to increase breastfeeding self‐efficacy among primiparous women

### 3.6 Onset, Dosage, Frequency, Duration of the Interventions and Follow‐Up Time Points

Intervention timing, dosage, frequency, duration and follow‐up varied across studies. Six interventions were delivered during the prenatal period only (Abbass‐Dick, Sun, et al. 2020; Aubel et al. 2004; Ingram and Johnson 2004; Raeisi et al. 2014; Su and Ouyang 2016; Wolfberg et al. 2004), five during the postnatal period only (Abbass‐Dick et al. 2015; Aidam et al. 2020; Johnston et al. 2017; Panahi et al. 2022; Pisacane et al. 2005), and eight across both periods (Bich et al. 2014, 2018; Bich and Cuong 2017; Gharaei et al. 2020; Ke et al. 2018; Kohan et al. 2019; Ozlüses and Celebioglu 2014; Rempel et al. 2020). Prenatal interventions were generally initiated between 25 and 39 weeks of gestation, although two studies did not specify the start time (Aubel et al. 2004; Wolfberg et al. 2004). Postnatal interventions began between 2 days and 1 month postpartum, with two studies not reporting the onset time (Aidam et al. 2020; Pisacane et al. 2005).

Intervention frequency ranged from one to six sessions, with additional formats including daily, monthly, fortnightly, or continuous or ongoing online delivery (Abbass‐Dick, Sun, et al. 2020; Aidam et al. 2020; Johnston et al. 2017; Ozlüses and Celebioglu 2014; Wolfberg et al. 2004). Session duration ranged from 5 min to 3 h, most commonly 30–45 min (Bich et al. 2014, 2018; Ingram and Johnson 2004; Ke et al. 2018; Panahi et al. 2022; Pisacane et al. 2005).

Follow‐up duration ranged from 2 days to 52 weeks postpartum, and 11 studies assessed exclusive breastfeeding at 6 months postpartum.

### 3.7 Delivery of the Interventions

Interventions were delivered using five formats, with more than half employing multiple formats (*n* = 11) and eight using a single format. These included: (1) face‐to‐face education and counselling (*n* = 16), such as group or individual sessions and home visits; (2) media and electronic resources (*n* = 12), including mass media, videos, websites, online platforms, and e‐health; (3) community and group activities (*n* = 6), such as group classes, fathers' groups, and community events; (4) technology demonstrations (*n* = 3), using aids such as breast models and feeding equipment; and (5) distance support (*n* = 2), including telephone, text, and video‐based counselling.

Most interventions were delivered by researchers (*n* = 8) or community and healthcare workers (*n* = 8), including community health workers (*n* = 5), midwives (*n* = 2), or both (*n* = 1). A small number were delivered by NGO staff, lactation specialists, or did not specify implementers (*n* = 3). Most implementers received professional training.

### 3.8 Outcome Measures of the Interventions

Exclusive breastfeeding rate was the primary outcome across all 19 studies. The WHO defines exclusive breastfeeding as feeding infants only breast milk, without any additional food or liquid except prescribed medicines, vitamins, or minerals within the previous 24 h (Bich et al. 2018). Secondary outcomes included breastfeeding self‐efficacy, infant feeding attitudes, partner and family support, breastfeeding knowledge, breastfeeding difficulties, parental relationships, and infant development. Detailed outcome measurement tools are summarised in Table 3.

**TABLE 3:** Measurements of intervention outcomes (*n* = 19).

Authors, year	Evaluation of exclusive breastfeeding rate	Evaluation of knowledge and attitudes on breastfeeding	Evaluation of the parental relationship	Evaluation of perceptions of breastfeeding and breastfeeding support	Evaluation of intervention use	Infant development
Pisacane et al. 2005	Questionnaire recommended by the WHO	NA	NA	Self‐constructed questionnaire on breastfeeding help received from partners	NA	NA
Ingram and Johnson 2004	Peer report	NA	NA	Self‐constructed questionnaire on perceived level of emotional and practical support for mothers from the prenatal to postnatal periods	NA	NA
Raeisi et al. 2014	Self‐constructed questionnaire about post‐partum characteristics	Self‐constructed questionnaire on parents' awareness based on consistent studies by five breastfeeding experts from the Tehran University of Medical Sciences	NA	NA	NA	NA
Aidam et al. 2020	Self‐constructed questionnaire	Self‐constructed questionnaire on maternal and child health and nutrition knowledge, attitudes and practices. Self‐constructed questionnaire on infant and young child feeding knowledge, beliefs and practices.	NA	NA	NA	NA
Bich and Cuong 2017	Structured interview	Structured interview of 22 items from the recommendations of the WHO	NA	Self‐constructed questionnaire to measure the knowledge, attitudes and practice of fathers' involvement in women and child care	NA	NA
Bich et al. 2018	Self‐constructed questionnaire	NA	NA	NA	NA	NA
Abbass‐Dick et al. 2015	Follow‐up data questionnaires	Iowa Infant Feeding Attitude Scale (IIFAS)	Coparenting Relationship Scale (CRS)	Postpartum Partner Support Scale (PPSS). Breastfeeding Self‐Efficacy Scale–Short Form (BSES‐SF).	A 4‐point self‐constructed scale	NA
Wolfberg et al. 2004	Self‐report	NA	NA	NA	NA	NA
Kohan et al. 2019	Self‐constructed questionnaire	NA	NA	Self‐constructed questionnaire on women's breastfeeding empowerment	NA	NA
Su and Ouyang 2016	Telephone interview	Breastfeeding Knowledge Scale (BKS). Iowa Infant Feeding Attitude Scale (IIFAS).	NA	NA	NA	NA
Ozlüses and Celebioglu 2014	Peer report	NA	NA	Paternal‐Infant Attachment Scale (PIAS)	NA	NA
Panahi et al. 2022	Self‐constructed questionnaire about ‘Exclusive Breastfeeding Status’ and an observational checklist	NA	NA	Self‐constructed questionnaire to assess ‘Fathers' support for Breastfeeding’	NA	NA
Ke et al. 2018	Self‐constructed questionnaire	Iowa Infant Feeding Attitude Scale (IIFAS). Breastfeeding Knowledge Questionnaire (BKQ).	NA	Breastfeeding Family Support Questionnaire (BFSQ)	NA	NA
Bich et al. 2014	Self‐constructed questionnaire	NA	NA	NA	NA	NA
Rempel et al. 2020	Face‐to‐face interview	NA	A 16‐item self‐report of Parental Relationship Quality developed by Gere and MacDonald	Partner Breastfeeding Influence Scale (PBIS)	NA	Developmental Milestones Checklist–II (at 9 months postpartum)
Johnston et al. 2017	Peer report	NA	Self‐constructed questionnaire of parental psychosocial well‐being	Self‐constructed questionnaire on postpartum depression, parenting experience, anxiety, social support and stress	NA	NA
Aubel et al. 2004	Individual interview	Individual interview on nutritional knowledge and advice	NA	Focus group interview on changes in the advice/practices of husbands, grandmothers, and mothers regarding women's and children's nutrition	NA	NA
Abbass‐Dick, Sun, et al. 2020	Peer report	Comprehensive Breastfeeding Knowledge Scale (CBKS). Iowa Infant Feeding Attitude Scale (IIFAS).	A 15‐item self‐constructed questionnaire	Breastfeeding Self‐Efficacy Scale‐Short Form (BSES‐SF). Postpartum Partner Support Scale (PPSS).	‐Interview to collect data on the use of eHealth resources: breastfeeding resource use and intervention use	NA
Gharaei et al. 2020	Telephone interview	NA	NA	Breastfeeding self‐efficacy scale‐short form (BSES‐SF). Basic questionnaire on husbands' support to the mothers during childcare activities.	NA	NA

#### 3.8.1 Primary Outcomes

Post‐intervention exclusive breastfeeding rates or breastfeeding duration were reported in all 19 studies.

##### 3.8.1.1 Effects of Family Breastfeeding Interventions on Exclusive Breastfeeding Rates at 1, 4 and 6 Months Postpartum

**FIGURE 4:**
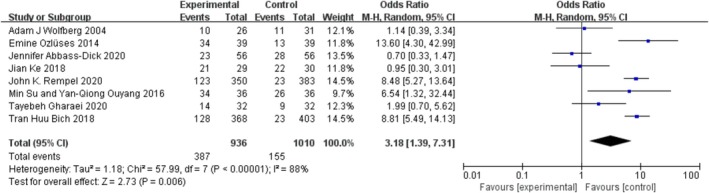
Effect of family breastfeeding interventions on exclusive breastfeeding rates at 1 month.

**FIGURE 5:**
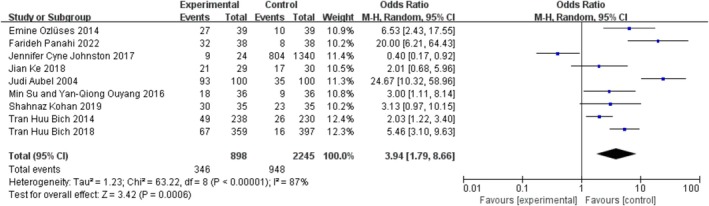
Effect of family breastfeeding interventions on exclusive breastfeeding rates at 4 months.

**FIGURE 6:**
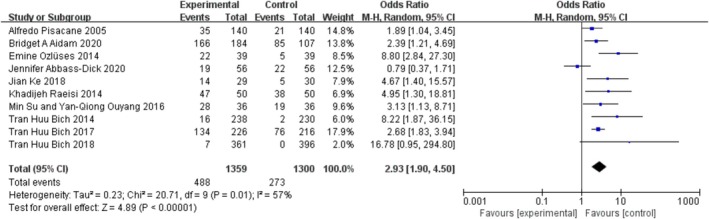
Effect of family breastfeeding interventions on exclusive breastfeeding rates at 6 months.

Out of 19 studies, eight reported exclusive breastfeeding rates at 1 month postpartum, nine at 4 months, and ten at 6 months (Figures 4, 5, 6). Compared with controls, exclusive breastfeeding rates were significantly higher in the intervention group at 1 month (OR = 3.18, 95% CI [1.39, 7.31], *p* = 0.006), 4 months postpartum (OR = 3.94, 95% CI [1.79, 8.66], *p* < 0.001), and 6 months postpartum (OR = 2.93, 95% CI [1.90, 4.50], *p* < 0.001).

**FIGURE 7:**
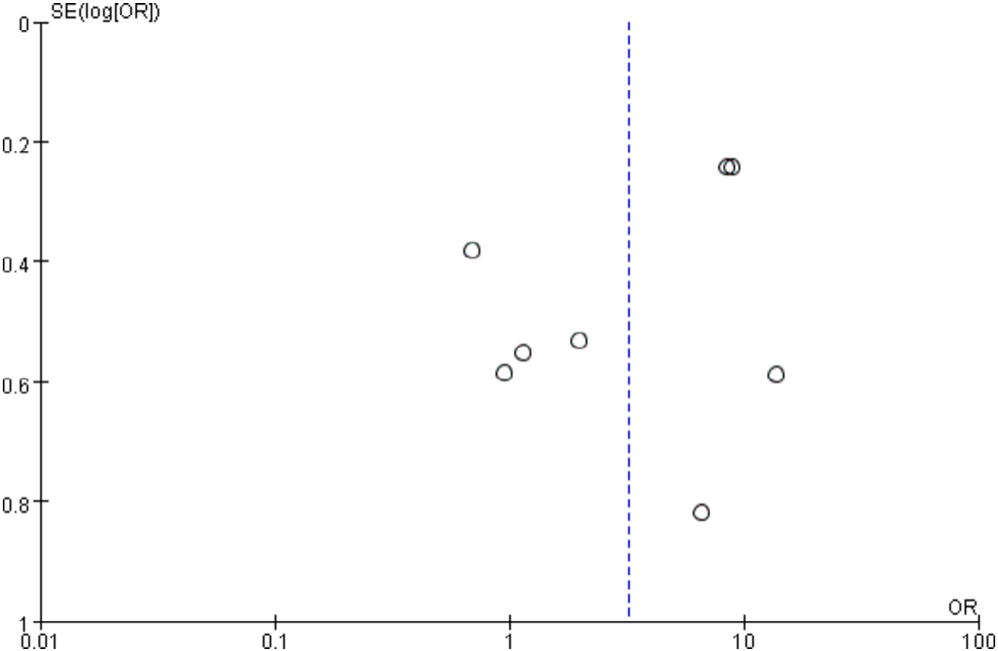
Funnel plot of the exclusive breastfeeding rates at 1 month.

**FIGURE 8:**
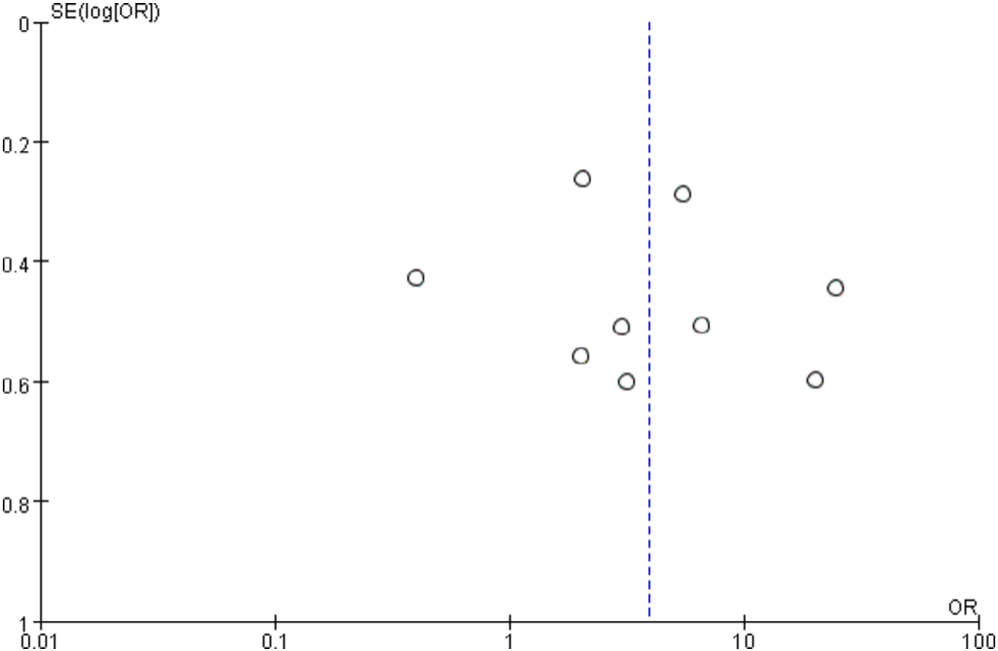
Funnel plot of the exclusive breastfeeding rates at 4 months.

**FIGURE 9:**
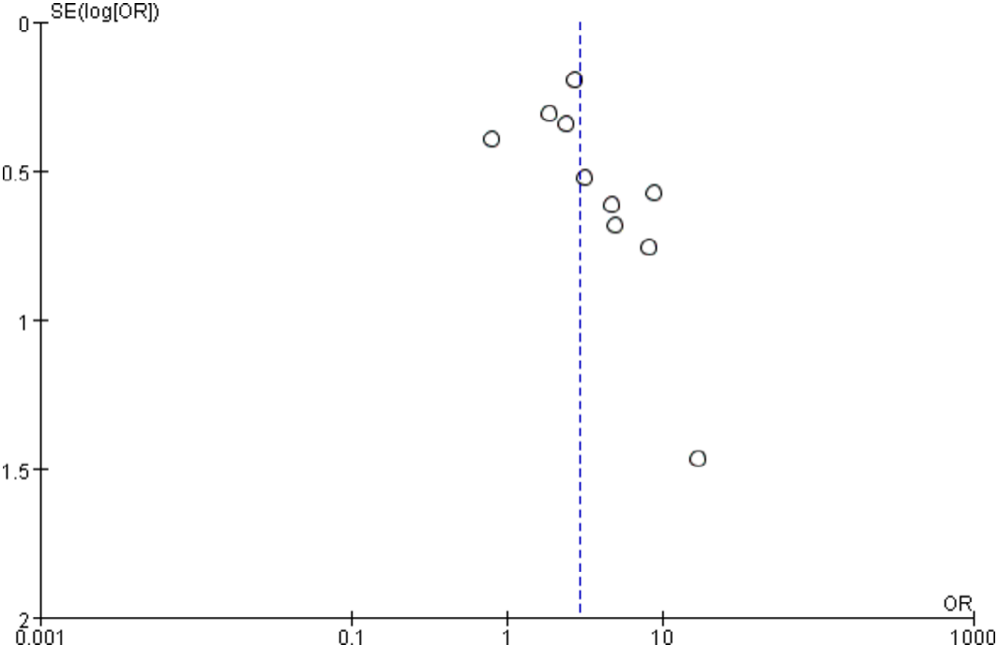
Funnel plot of the exclusive breastfeeding rates at 6 months.

Substantial heterogeneity was observed at 1 month (*I* ^2^ = 88%, *p* < 0.001), 4 months (*I* ^2^ = 87%, *p* < 0.001), and moderate heterogeneity at 6 months (*I* ^2^ = 57%, *p* < 0.001). Results from fixed‐ and random‐effects models were consistent. Funnel plots appeared symmetric, suggesting no evidence of publication bias (Figures 7, 8, 9). Sensitivity analyses at 1 and 4 months indicated stable pooled estimates, suggesting that heterogeneity was likely attributable to differences in intervention characteristics or participant populations.

At 6 months postpartum, exclusion of Abbass‐Dick, Sun, et al.'s (2020) study reduced heterogeneity (OR = 3.18, 95% CI [2.26, 4.47], *p* < 0.001, *I* ^2^ = 26%), indicating its contribution to variability. However, the pooled effect size and statistical significance remained unchanged, supporting the robustness of the overall findings.

##### 3.8.1.2 Effects of Different Intervention Participants on Exclusive Breastfeeding Rates at 6 Months

**FIGURE 10:**
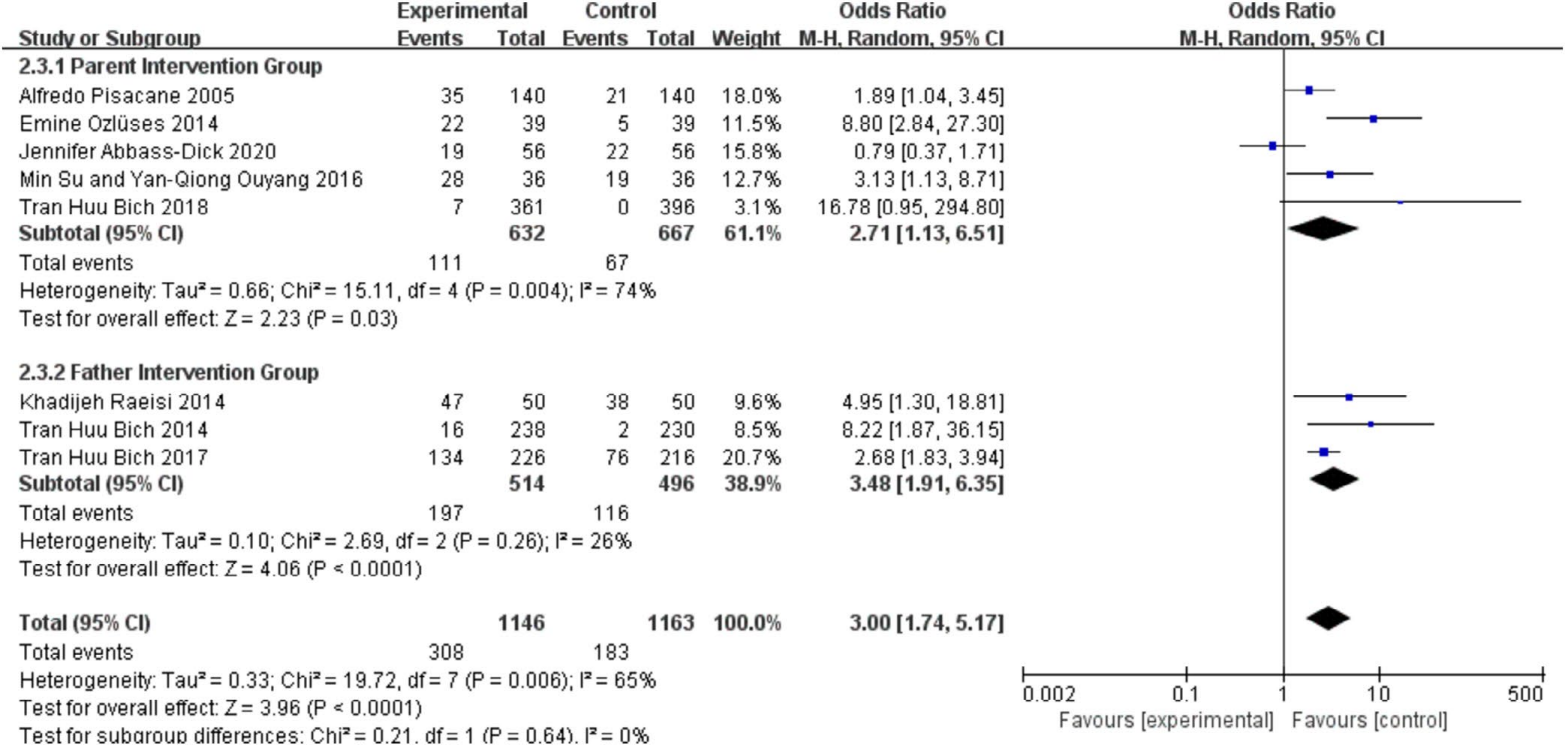
Effect of family breastfeeding interventions on exclusive breastfeeding rates at 6 months for different family member groups.

Based on the different study populations in the 19 studies, we divided the studies into the following four intervention groups: parent, father, mother and main family member, and grandmother. Due to the limited number of studies, only the parent intervention group and the father intervention group were included in the subgroup analysis to explore the impact on exclusive breastfeeding rates at 6 months (Figure 10). Five studies included both parents in their intervention (Abbass‐Dick, Sun, et al. 2020; Bich et al. 2018; Ozlüses and Celebioglu 2014; Pisacane et al. 2005; Su and Ouyang 2016), involving 1299 pairs of parents. The father intervention group was the focus of three studies (Bich et al. 2014; Bich and Cuong 2017; Raeisi et al. 2014), involving 1037 fathers. Overall, the meta‐analysis showed that the intervention significantly increased the exclusive breastfeeding rates of mothers in both the parent intervention group (OR = 2.71, 95% CI [1.13, 6.51], *p* = 0.03) and the father intervention group at 6 months (OR = 3.48, 95% CI [1.91, 6.35], *p* < 0.001), compared to those in the control group (OR = 3.00, 95% CI [1.74, 5.17], *p* < 0.001).

##### 3.8.1.3 Impacts of Different Theoretical Frameworks on Exclusive Breastfeeding Rates Within 2 Months Postpartum

**FIGURE 11:**
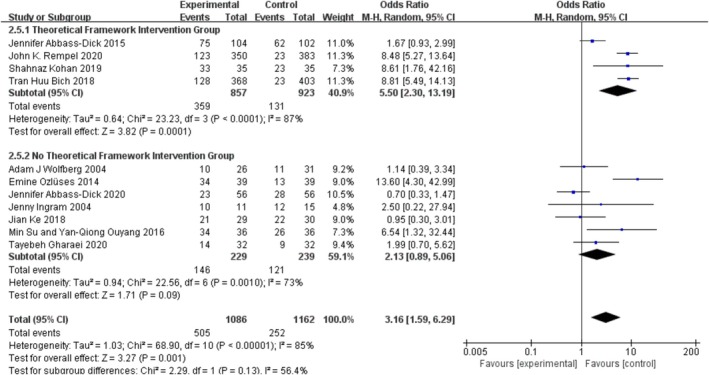
Impact of different theoretical frameworks on exclusive breastfeeding rates within 2 months postpartum.

Among the 19 studies, five utilised a theoretical framework that guided the development of their intervention, while the remaining 14 did not mention a theoretical framework. Eleven studies assessed mothers' exclusive breastfeeding rates within 2 months postpartum; these were included in the meta‐analysis and divided into intervention groups with and without a theoretical framework. Four studies were included in the theoretical framework intervention group, involving 1819 families (Abbass‐Dick et al. 2015; Bich et al. 2018; Kohan et al. 2019; Rempel et al. 2020). Seven studies were included in the no theoretical framework intervention group, involving 617 families (Abbass‐Dick, Sun, et al. 2020; Gharaei et al. 2020; Ingram and Johnson 2004; Ke et al. 2018; Ozlüses and Celebioglu 2014; Su and Ouyang 2016; Wolfberg et al. 2004). Overall, the meta‐analysis found that the intervention significantly increased the exclusive breastfeeding rates of mothers within 2 months postpartum in both intervention groups compared to the controls (OR = 3.16 [1.59, 6.29], *p* = 0.001). However, while the sub‐group meta‐analysis showed that the theoretical framework intervention group reported a significant improvement in exclusive breastfeeding rates within 2 months postpartum compared to those in the control group (OR = 5.50, 95% CI [2.30, 13.19], *p* = 0.000), those in the non‐theoretical framework intervention group did not achieve a statistically significant increase in the mothers' exclusive breastfeeding rates compared to the controls (OR = 2.13, 95% CI [0.89, 5.06], *p* = 0.09) (Figure 11).

##### 3.8.1.4 Effects of Different Intervention Delivery Formats on Exclusive Breastfeeding Rates at 6 Months Postpartum

The 19 studies were classified into five different types according to their intervention delivery formats. Eight used only one format, with six of these using face‐to‐face education and counselling (Aubel et al. 2004; Gharaei et al. 2020; Johnston et al. 2017; Ozlüses and Celebioglu 2014; Panahi et al. 2022; Raeisi et al. 2014). The other two studies used social media and electronic resources (Abbass‐Dick, Sun, et al. 2020; Ingram and Johnson 2004). The remaining 11 studies employed a combination of more than one intervention delivery format, including face‐to‐face education and counselling (*n* = 10) (Abbass‐Dick et al. 2015; Aidam et al. 2020; Bich et al. 2014; Bich and Cuong 2017; Ke et al. 2018; Kohan et al. 2019; Pisacane et al. 2005; Rempel et al. 2020; Su and Ouyang 2016; Wolfberg et al. 2004), media and electronic resources (*n* = 10) (Abbass‐Dick et al. 2015; Bich et al. 2014, 2018; Bich and Cuong 2017; Ke et al. 2018; Kohan et al. 2019; Pisacane et al. 2005; Rempel et al. 2020; Su and Ouyang 2016; Wolfberg et al. 2004), community and group activities (*n* = 6) (Aidam et al. 2020; Bich et al. 2014, 2018; Bich and Cuong 2017; Kohan et al. 2019; Rempel et al. 2020), technology demonstrations (*n* = 3) (Rempel et al. 2020; Su and Ouyang 2016; Wolfberg et al. 2004), and distance support (*n* = 2) (Bich et al. 2014; Kohan et al. 2019).

**FIGURE 12:**
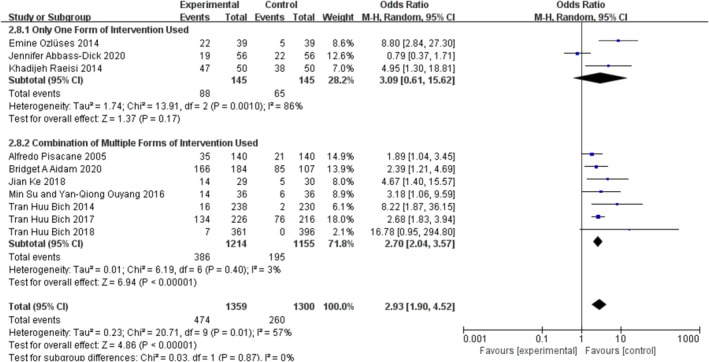
Effect of different forms of intervention on the rates of exclusive breastfeeding at 6 months of age.

A sub‐group meta‐analysis was conducted separately to compare the effects of the intervention on mothers' exclusive breastfeeding rates at 6 months postpartum in terms of using one intervention format and multiple formats compared to those in the controls (Figure 12). The meta‐analysis showed no statistical significance on the exclusive breastfeeding rates of mothers at 6 months postpartum between the one intervention format group and the control group (OR = 3.09, 95% CI [0.61, 15.62], *p* = 0.17), while the group using multiple intervention methods showed a significant increase in mothers' exclusive breastfeeding rates at 6 months postpartum compared to the controls (OR = 2.70, 95% CI [2.04, 3.57], *p* < 0.001).

##### 3.8.1.5 Effects of Different Intervention Onsets on Mothers' Exclusive Breastfeeding Rates at 4–6 Months Postpartum

**FIGURE 13:**
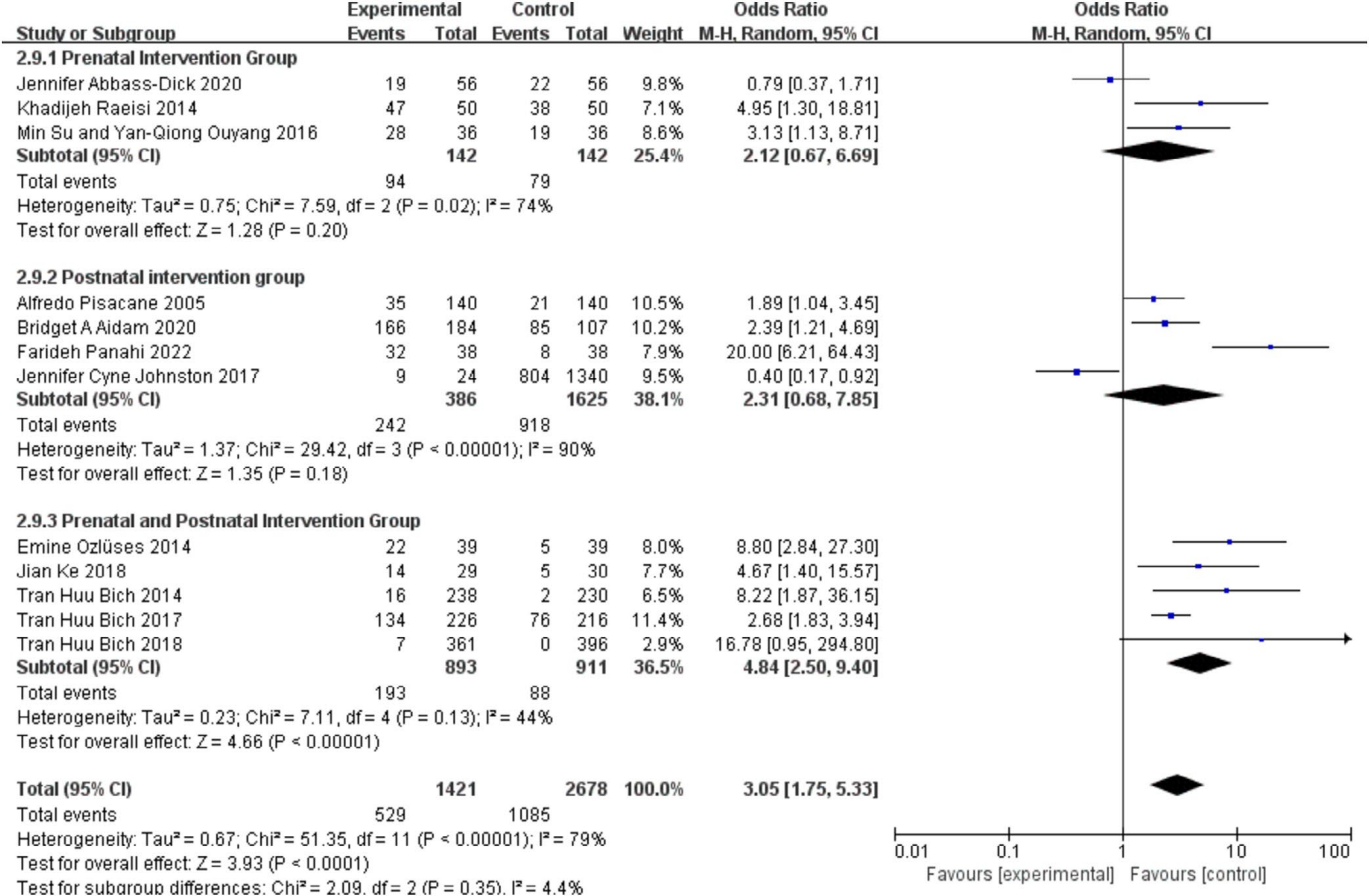
Comparison of the effect of different intervention time groups on the rates of exclusive breastfeeding from 4 to 6 months of age.

Of the 19 studies, the breastfeeding interventions in six studies were conducted during pregnancy, five after childbirth, and eight both prenatally and postnatally. A meta‐analysis was conducted to compare the effects of different intervention onsets on mothers' exclusive breastfeeding rates at 4–6 months postpartum (Figure 13). The meta‐analysis showed that neither the prenatal intervention group (OR = 2.12, 95% CI [0.67, 6.69], *p* = 0.2) nor the postnatal intervention group (OR = 2.31, 95% CI [0.68, 7.85], *p* = 0.18) showed significant overall effects compared to the control group. By contrast, the group with the intervention covering both the prenatal and postnatal periods demonstrated a significant positive effect on mothers' exclusive breastfeeding rates compared to the control group (OR = 4.84, 95% CI [2.50, 9.40], *p* < 0.001).

#### 3.8.2 Secondary Outcomes

##### 3.8.2.1 Breastfeeding Self‐Efficacy

Three studies measured the breastfeeding self‐efficacy of fathers and mothers using the Breastfeeding Self‐Efficacy Scale‐Short Form (BSES‐SF) (Abbass‐Dick et al. 2015; Abbass‐Dick, Sun, et al. 2020; Dennis 2003; Gharaei et al. 2020). These studies examined breastfeeding self‐efficacy from 2 weeks postpartum to 8 weeks postpartum and involved 495 families. In Abbass‐Dick et al.'s (2015) study, no significant difference in breastfeeding self‐efficacy scores was reported between fathers in the intervention and control groups (intervention group: 55.9 ± 8.4 versus control group: 53.1 ± 11.2, *p* = 0.06) at 6 weeks postpartum. But the breastfeeding self‐efficacy of fathers in the intervention group increased significantly compared to the control at 6 weeks postpartum (intervention group: before 48.5 ± 9.7, after 55.9 ± 8.4 versus control group: before 49.5 ± 9.9, after 53.1 ± 11.2, *p* = 0.03). In Gharaei's study (Gharaei et al. 2020), the breastfeeding self‐efficacy of mothers in the intervention group with grandmother involvement was compared with that of the control group without grandmother involvement. The results showed that mothers in the intervention group had significantly higher breastfeeding self‐efficacy than the control group at 4 weeks postpartum (intervention group: 61.71 ± 2.66 versus control group: 56.62 ± 9.12, *p* = 0.004). This trend continued at 8 weeks postpartum (intervention group: 63.68 ± 2.14 versus control group: 60.03 ± 6.32, *p* = 0.003). However, Abbass‐Dick, Sun, et al.'s (2020) study found no significant difference between the intervention and control groups in the breastfeeding self‐efficacy of mothers.

##### 3.8.2.2 Infant Feeding Attitude

Three studies, involving 348 families, used the Iowa Infant Feeding Attitude Scale (IIFAS) (de la Mora et al. 1999) to assess parents' attitudes towards breastfeeding (Abbass‐Dick, Sun, et al. 2020; Ke et al. 2018; Su and Ouyang 2016). In Su and Ouyang's (2016) study, both mothers (before: 61.50 versus after: 69.00, *p* < 0.000) and fathers (before: 59.14 versus after: 66.50, *p* < 0.000) in the intervention group showed a significant improvement in their IIFAS scores after the intervention. There was also a significant increase in the IIFAS scores of mothers in the intervention group compared to mothers in the control group (intervention group: 7.50 versus control group: 5.17, *p* = 0.001); whereas in Ke et al.'s (2018) study and Abbass‐Dick, Sun, et al.'s (2020) study, there was no significant difference between the intervention and control groups.

##### 3.8.2.3 Partner and Family Support

Nine of the 19 studies assessed partner and family support using validated or self‐developed instruments at various time points. The Postpartum Partner Support Scale (PPSS) (Dennis and Ross 2006) was applied in two studies (Abbass‐Dick et al. 2015; Abbass‐Dick, Sun, et al. 2020). Abbass‐Dick et al.'s (2015) study reported that more mothers in the intervention group received breastfeeding assistance from fathers within the first 6 weeks postpartum (intervention group: 71% versus control group: 52%, *p* = 0.02) and expressed greater satisfaction with paternal involvement (intervention group: 89% versus control group: 78.1%, *p* = 0.04). Their 2020 study also demonstrated significantly higher partner support scores in the intervention group at 4, 12, and 52 weeks postpartum.

One study using the Breastfeeding Family Support Questionnaire (BFSQ) reported consistently higher perceived family support in the intervention group from 1 to 6 months postpartum (*p* < 0.05) (Ke et al. 2018; Zhu et al. 2013). Similarly, Rempel et al. (2020), using the Partner Breastfeeding Influence Scale (PBIS), reported significantly higher paternal support scores in the intervention group at both 1 and 4 months postpartum, particularly in the domains of helpfulness and responsiveness (Rempel et al. 2020; Rempel et al. 2017). Specifically, scores for helping and responsiveness were significantly higher in the intervention group than in the control group at both follow‐up time points (helping at 1 month: 3.09 ± 0.55 vs. 2.94 ± 0.59, *p* = 0.02; responsiveness at 1 month: 2.90 ± 0.54 vs. 2.80 ± 0.57, *p* = 0.01; helping at 4 months: 2.84 ± 0.61 vs. 2.74 ± 0.70, *p* = 0.01; responsiveness at 4 months: 2.87 ± 0.54 vs. 2.76 ± 0.61, *p* = 0.01).

Four additional studies using self‐designed questionnaires also reported significantly greater partner and family support in the intervention group across follow‐up periods ranging from 2 weeks to 12 months postpartum (*p* < 0.05) (Kohan et al. 2019; Panahi et al. 2022; Pisacane et al. 2005; Raeisi et al. 2014). Qualitative findings from Su and Ouyang (Su and Ouyang 2016) further indicated that partners in the intervention group were more actively involved in infant care, household tasks and emotional support.

##### 3.8.2.4 Breastfeeding Knowledge

Three of the included studies measured parents' breastfeeding knowledge before and after the intervention.

The Breastfeeding Knowledge Scale (Ouyang et al. 2012) was used to assess mothers' level of breastfeeding knowledge in a study by Su and Ouyang (Su and Ouyang 2016). Mothers in the intervention group scored significantly higher than the controls in terms of breastfeeding knowledge (intervention group: 19.75 versus control group: 14.81, *p* = 0.009).

One study used the Comprehensive Breastfeeding Knowledge Scale (CBKS) to assess parents' breastfeeding knowledge at 2 and 4 weeks postpartum (Abbass‐Dick, Newport, et al. 2020; Abbass‐Dick, Sun, et al. 2020). No significant difference in breastfeeding knowledge was reported between the intervention and control groups after the intervention.

Bich and Cuong's (2017) study used 22 items recommended by the WHO to measure fathers' knowledge, attitudes and practices regarding exclusive breastfeeding (Bich and Cuong 2017). The study reported that fathers in the intervention group had significantly higher breastfeeding knowledge scores than those in the control group (intervention group: 25.8 ± 6.4 versus control group: 19.5 ± 6.6, *p* < 0.001).

##### 3.8.2.5 Other Relevant Results

The Paternal‐Infant Attachment Scale (PIAS) was utilised to assess parent‐infant attachment in Ozlüses's study (Condon et al. 2008; Ozlüses and Celebioglu 2014). It was found that the father‐infant attachment scores of the intervention group were higher than those of the control group (intervention group: 89.51 ± 7.05 versus control group: 82.37 ± 12.80, *p* < 0.001). In addition, the provision of breastfeeding education to fathers significantly enhanced father‐infant attachment.

The Co‐parenting Relationship Scale (CRS) was used in one study to assess the co‐parenting relationship at 6 and 12 weeks postpartum (Abbass‐Dick et al. 2015; Feinberg et al. 2012). However, no significant differences between the intervention and control groups were reported in co‐parenting relationship scores.

In Rempel et al.'s study, the developmental status of infants at 9 months of age was assessed using the Developmental Milestones Checklist‐II (DMCC‐II) (Rempel et al. 2020). It was found that infants in the intervention group had significantly higher motor, language, and personal‐social development scores than those in the control group at 9 months (*p* < 0.001). It was also reported that fathers' helping behaviours significantly predicted infants' motor and personal‐social development (*p* < 0.05).

## 4 Discussion

This systematic review included 19 studies published across diverse settings and time periods, reflecting increasing research attention to the role of family involvement in breastfeeding outcomes.

### 4.1 Exclusive Breastfeeding Rates

#### 4.1.1 Intervention Participants

The meta‐analysis showed that family‐based interventions significantly increased exclusive breastfeeding rates at 6 months postpartum. Subgroup analysis indicated that both parent‐focused and father‐only interventions were effective, suggesting that paternal involvement supports maternal breastfeeding practices and facilitates adjustment to new family roles. Fathers provide both informational support, through practical guidance, and emotional support, including encouragement and attentive listening, which may help families adapt more effectively (Kohn et al. 2012; Rempel et al. 2017). These findings highlight the importance of actively involving fathers in future interventions.

Only a small number of studies involved grandparents (Aidam et al. 2020; Aubel et al. 2004; Gharaei et al. 2020; Ingram and Johnson 2004), and their heterogeneity precluded meta‐analysis of their effects on exclusive breastfeeding. However, individual studies consistently reported improvements in grandparents' knowledge and attitudes towards infant feeding following intervention (Aubel et al. 2004; Kohan et al. 2019). This finding suggests that grandparents may represent an important but underexplored target for breastfeeding interventions (Sear and Coall 2011). In settings where grandparents are actively involved in childcare and infant feeding decisions, improving grandparents' breastfeeding knowledge and support behaviours may enhance the family environment for breastfeeding and contribute to more favourable breastfeeding outcomes (Aidam et al. 2020; Aubel et al. 2021).

The potential influence of grandparents may also vary across countries and cultural contexts. In many Asian countries, multigenerational co‐residence and active grandparental caregiving are relatively common, whereas childcare responsibilities are more often concentrated within the nuclear family in many Western countries (Price et al. 2018; Sear and Coall 2011). In addition, the breastfeeding support environment differs across settings. Several included studies were conducted in Baby‐Friendly Hospital Initiative (BFHI) settings, where mothers and families may already receive structured breastfeeding education and professional support (Fan et al. 2025). Some studies also identified maternal employment and return to work as barriers to breastfeeding (Rollins et al. 2016). Therefore, differences in family structures, healthcare support environments, and caregiving arrangements may partly explain variations in intervention effectiveness across studies and should be considered when interpreting and generalising the findings (Aubel 2021).

#### 4.1.2 Intervention Theoretical Frameworks

This systematic review and meta‐analysis found that theoretically informed breastfeeding interventions were more effective in improving exclusive breastfeeding rates than non‐theoretical approaches, consistent with previous findings (Chipojola et al. 2020; Wong et al. 2021). This may be because non‐theoretical interventions primarily focus on practical breastfeeding knowledge and skills, such as feeding techniques, family support and management of common problems (Okhovat et al. 2023). In contrast, theory‐based interventions not only provide practical knowledge but also address behavioural change mechanisms, family interaction, and collaborative support within the family context (Fan et al. 2022; Gau 2004; He et al. 2024; Huang et al. 2023).

The Theory of Planned Behaviour highlights the role of knowledge, beliefs, attitudes and values in shaping behavioural intentions, which in turn influence behaviour (Gau 2004; Huang et al. 2023). Social Cognitive Theory emphasises breastfeeding self‐efficacy, developed through performance accomplishments, vicarious experience, verbal persuasion and physiological feedback. Coparenting Theory underscores the importance of family cooperation and support in facilitating breastfeeding, particularly by enhancing fathers' capacity to recognise and address breastfeeding challenges (Feinberg 2002). Empowerment Theory‐based interventions aim to increase maternal autonomy and decision‐making through education and supportive networks (Kohan et al. 2019).

Similarly, interventions informed by Ecological Theory and Breastfeeding Team Theory emphasise the influence of interpersonal and environmental interactions and the importance of parental collaboration in breastfeeding support (Rempel et al. 2020; Zhao et al. 2024). These approaches have been associated with increased paternal support and longer exclusive breastfeeding duration.

These findings highlight the value of incorporating theoretical frameworks into intervention design. However, theoretical grounding alone does not guarantee effectiveness. One study reported no significant difference between theory‐based and non‐theory‐based interventions, suggesting that the relationship between theoretical models and behavioural outcomes remains complex (Fan et al. 2024). Further research is needed to compare different theoretical approaches and evaluate their applicability across diverse populations.

#### 4.1.3 Intervention Formats

Five main intervention formats were identified: face‐to‐face education and counselling, media and electronic resources, community and group activities, technical demonstrations, and remote support. These can be broadly categorised as online or offline approaches. None of the included studies directly compared their effectiveness, and most relied on traditional offline methods, with limited integration of both formats.

Online interventions, often delivered via mobile applications, offer flexibility and wider accessibility for family members seeking breastfeeding support (Dol et al. 2019; Wong and Chien 2023), whereas face‐to‐face approaches provide personalised interaction that can enhance maternal confidence and practical skills (Lee et al. 2016). Previous evidence suggests that prenatal education and websites are perceived as most useful during pregnancy, while websites and peer support are preferred postpartum (Abbass‐Dick, Sun, et al. 2020). Therefore, combining face‐to‐face education with online support may offer a more effective strategy for family‐centred breastfeeding interventions.

#### 4.1.4 Intervention Onset Time

Consistent with the findings of Galipeau (Galipeau et al. 2018), the timing of intervention onset varied considerably across the included studies. Subgroup analysis indicated that interventions spanning both prenatal and postnatal periods were more effective in improving exclusive breastfeeding than those delivered during a single period. One explanation may be that confidence gained through prenatal education declines after birth, limiting the long‐term effectiveness of prenatal‐only interventions (Babakazo et al. 2015; Kim et al. 2018). Postnatal interventions provide continued support by helping families recognise key breastfeeding issues and respond appropriately to maternal and infant care needs (Fan et al. 2024).

Therefore, combining prenatal and postnatal interventions appears more effective in sustaining exclusive breastfeeding for up to 6 months postpartum and improving overall breastfeeding outcomes (Dol et al. 2019; Kim et al. 2018; Wong et al. 2021).

### 4.2 Other Breastfeeding Outcomes

Beyond primary outcomes, this review examined secondary effects, including breastfeeding self‐efficacy, family support, father‐infant attachment and infant development. Intervention groups consistently reported improved breastfeeding self‐efficacy, indicating greater confidence in managing breastfeeding challenges (Gharaei et al. 2020). Family‐based interventions also enhanced partner and family support (Abbass‐Dick et al. 2015; Abbass‐Dick, Sun, et al. 2020; Rempel et al. 2020), and two studies reported improved breastfeeding knowledge (Bich and Cuong 2017; Su and Ouyang 2016). Additional benefits included improved father‐infant attachment and infant development (Ozlüses and Celebioglu 2014; Rempel et al. 2020).

Despite these findings, the limited number of studies warrants cautious interpretation. Increased paternal involvement may strengthen family support and parent–infant interactions. However, no significant improvements were observed in co‐parenting relationships, possibly reflecting complex family dynamics and the need for longer‐term interventions. Cultural and family factors may also influence outcomes (Abbass‐Dick et al. 2019; Davidson and Ollerton 2020). Future interventions should prioritise strengthening partner cooperation and communication. Overall, while individual‐level benefits were evident, relational and long‐term effects require further investigation.

### 4.3 Limitation

Several limitations should be acknowledged. The included studies varied in intervention content, theoretical frameworks, participant characteristics, and outcome measurements, which may limit the comparability of findings and contribute to heterogeneity across studies. Although some studies reported benefits of father and grandparent involvement, the limited number of interventions targeting grandparents and the lack of maternal‐only comparison groups restricted direct comparisons of the relative effectiveness of different family compositions. Most studies focused on short‐term breastfeeding outcomes, with limited evaluation of long‐term effects on breastfeeding continuation, co‐parenting relationships, and family dynamics. In addition, the combined effects of online and offline intervention delivery approaches remain insufficiently explored. Furthermore, although theoretical frameworks informed intervention design in several studies, the applicability and consistency of these frameworks across different family and cultural contexts require further evaluation. Moreover, the included studies were conducted in countries with diverse healthcare systems, parental leave policies, family structures, childcare arrangements, and cultural contexts. These contextual differences may have influenced intervention implementation and effectiveness and should be considered when interpreting and generalising the findings. Most included studies were also assessed as having a high or unclear risk of bias, particularly in relation to randomisation procedures and blinding; therefore, the findings should be interpreted with caution.

### 4.4 Implications for Nursing Practice

Nurses, midwives, lactation nurses, and public health nurses should actively involve fathers, grandparents, and other key family members in breastfeeding education and support across both antenatal and postnatal care. Integrating family‐centred breastfeeding interventions into routine nursing practice may help improve exclusive breastfeeding outcomes and strengthen family support for breastfeeding.

## 5 Conclusion

This study confirms the effectiveness of family‐based breastfeeding interventions in improving exclusive breastfeeding rates up to 6 months postpartum. These interventions also enhance maternal self‐efficacy, infant feeding attitudes, breastfeeding knowledge, partner and family support, father–infant attachment, and infant development. Engaging key family members and addressing breastfeeding needs across different stages may further optimise outcomes.

Future interventions should be guided by appropriate theoretical frameworks, span both prenatal and postnatal periods, and integrate offline and online delivery methods to meet diverse family needs. Greater inclusion of grandparents and other family members is also needed to inform more comprehensive and standardised models of family‐based breastfeeding support.

## Author Contributions

Z.X. was responsible for the conception of the study, designing the search strategies, study screening and selection, data extraction and analysis, as well as writing the original draft and revising the manuscript. X.Z. was responsible for data analysis and assisting with the revision of the search strategies. Y.Z. was responsible for data analysis. All authors read and approved the final manuscript. X.X. was responsible for conceptualising the study, data analysis, project administration, and writing and revising the manuscript.

## Funding

This study was supported by the Shenzhen Science and Technology Program [grant number: JCYJ20230807120312026, JCYJ20250604185253070], the nursing special project of Shenzhen Maternal and Child Healthcare Hospital [grant number: FYB2022003], and Sanming Project of Medicine in Shenzhen [grant number: SZSM202211032].

## Disclosure

No AI‐assisted technologies were used in the preparation of this manuscript.

## Ethics Statement

The authors have nothing to report.

## Consent

The authors have nothing to report.

## Conflicts of Interest

The authors declare no conflicts of interest.

## Supporting information


**Data S1:** PRISMA 2020 Checklist.

**Table S1:** Sociocultural and Breastfeeding Support Contexts of Included Studies (*n* = 19).

## Data Availability

The data that support the findings of this study are available from the corresponding author upon reasonable request.
