# Correction to ‘Family‐Centred Breastfeeding Interventions to Improve Exclusive Breastfeeding: A Systematic Review and Meta‐Analysis’

**DOI:** 10.1002/nop2.70782

**Published:** 2026-10-28

Xie, Z. , X. Zhang , Y. Zhang , and X. Xiao . 2026. “Family‐Centred Breastfeeding Interventions to Improve Exclusive Breastfeeding: A Systematic Review and Meta‐Analysis.” Nursing Open 13, no. 7: e70669. 10.1002/nop2.70669.PMC1333232742400223

The first author's affiliations have been updated and the affiliation list order has been re‐ordered in this paper since it was first published.

In the originally published version, the author–affiliation information was listed in the following order:

Ziyang Xie^1^, Xuanli Zhang^1^, Yunyi Zhang^1^, Xiao Xiao^1,2^

1. School of Nursing, Southern Medical University, Guangzhou, China

2. Outpatient and Emergency Department, Shenzhen Maternity and Child Healthcare Hospital, Southern Medical University, Shenzhen, China

The correct author–affiliation information is:

Ziyang Xie^1,2^, Xuanli Zhang^2^, Yunyi Zhang^2^, Xiao Xiao^1,2^

1. Outpatient and Emergency Department, Shenzhen Maternity and Child Healthcare Hospital, Southern Medical University, Shenzhen, China

2. School of Nursing, Southern Medical University, Guangzhou, China

We apologise for this error.

