# Supplementary material for: Family‐Centred Breastfeeding Interventions to Improve Exclusive Breastfeeding: A Systematic Review and Meta‐Analysis
Source: Nurs Open. 2026 Jul 3;13(7):e70669. doi: 10.1002/nop2.70669 (PMC13332327; doi:10.1002/nop2.70669)
Supplement: Supplementary file 2 — Table S1: Sociocultural and Breastfeeding Support Contexts of Included Studies (n = 19). [file NOP2-13-e70669-s002.docx]

**Supplementary Table S1. Sociocultural and Breastfeeding Support Contexts of Included Studies(n=19)**

| **Study** | **Country** | **Study Setting** | **Breastfeeding Support Setting** | **Family Members Targeted** | **Sociocultural Characteristics Relevant to Breastfeeding** | **Community Support Component** | **Employment / Return-to-Work Factors Reported** |
| --- | --- | --- | --- | --- | --- | --- | --- |
| Pisacane et al., 2005 | Italy | University obstetric department | BFHI hospital-based breastfeeding support and paternal training. | Fathers | Fathers were recognised as important sources of breastfeeding support. | No | Maternal return to work identified as a breastfeeding barrier. |
| Ingram & Johnson, 2004) | England | Community health centre and family homes | Community-based antenatal family-support intervention. | Fathers and grandmothers | Strong family networks and matriarchal influences within socioeconomically disadvantaged communities. | Yes | NA |
| Raeisi et al., 2014 | Iran | Hospital / family health research centre | Hospital-linked prenatal breastfeeding training for fathers. | Fathers | Family support considered important for breastfeeding continuation. | No | NA |
| Aidam et al., 2020 | The Republic of Sierra Leone | Rural communities | Community maternal and child nutrition programme. | Grandmothers | Grandmothers and older women traditionally served as infant-feeding advisers and caregivers. | Yes | NA |
| Bich & Cuong, 2017 | Vietnam | Rural commune health centres | Community-based intervention integrated into local health services. | Fathers | Traditional beliefs, family pressure, and cultural barriers influenced feeding decisions. | Yes | Maternal employment and early return to work identified as barriers. |
| Bich et al., 2018 | Vietnam | Community-based districts / commune health centres | Routine maternal-child health services. | Fathers | Family, community, and social norms influenced breastfeeding practices. | Yes | Maternal workload and employment discussed. |
| Abbass-Dick et al., 2015 | Canada | Large teaching hospital | Standard hospital and community breastfeeding support. | Fathers | Conducted in a culturally and socioeconomically diverse urban population. | No | NA |
| Wolfberg et al., 2004 | America | University obstetric practice | Hospital-based prenatal father education. | Fathers | Low breastfeeding prevalence among minority and low-income populations highlighted. | No | NA |
| Kohan et al., 2019 | Iran | Comprehensive health centres | Breastfeeding empowerment programme delivered through health centres. | Mothers, fathers, and family members | Family support viewed as a central component of breastfeeding empowerment. | No | NA |
| Su & Ouyang, 2016 | China | Baby-Friendly university hospital | BFHI hospital-based breastfeeding education. | Fathers | Father involvement emphasised within the Chinese family caregiving context. | No | Maternal return to work identified as a barrier. |
| Ozlüses & Celebioglu, 2014 | Turkey | State maternity hospital | Hospital-based breastfeeding education before discharge. | Fathers | Paternal involvement and father–infant attachment emphasised. | No | Not reported. |
| Panahi et al., 2022 | Iran | Health centre | BFHI-related father education and counselling programme. | Fathers | Islamic cultural values supportive of breastfeeding were described. | No | NA |
| Ke et al., 2018 | China | Government-funded Baby-Friendly tertiary hospital | BFHI tertiary hospital care plus post-discharge follow-up support. | Fathers and grandmothers | Multigenerational co-residence and grandparent involvement in childcare were common. | No | NA |
| Bich et al., 2014 | Vietnam | Commune health centres | Community maternal-child health services. | Fathers | Rural family structures and family support played important roles in breastfeeding decisions. | Yes | Maternal employment and workload identified as barriers. |
| Rempel et al., 2020 | Vietnam | Community-based fathering programme | Community intervention using health workers and local resources. | Fathers | Transition from traditional paternal roles toward co-parenting and shared caregiving. | Yes | NA |
| Johnston et al., 2017 | Canada | Public health well-child clinics | Nurse-led public health breastfeeding support services. | Parents | No specific sociocultural context reported. | Yes | NA |
| Aubel et al., 2004 | Italy | Rural villages | Community nutrition education programme. | Grandmothers | Grandmothers were key decision-makers regarding maternal and infant feeding practices. | Yes | NA |
| Abbass-Dick et al., 2020b | Canada | Community and online eHealth intervention | Community resources and online breastfeeding support. | Mothers and co-parents | NA | Yes | NA |
| Gharaei et al., 2020 | Iran | Antenatal clinic / hospital | Hospital-based antenatal and postpartum breastfeeding education. | Maternal grandmothers | Maternal grandmothers traditionally provided postpartum support and infant care. | No | Fathers’ work commitments limited participation. |
